# Detection and characterisation of coronaviruses in migratory and non-migratory Australian wild birds

**DOI:** 10.1038/s41598-018-24407-x

**Published:** 2018-04-13

**Authors:** Anthony Chamings, Tiffanie M. Nelson, Jessy Vibin, Michelle Wille, Marcel Klaassen, Soren Alexandersen

**Affiliations:** 1 Geelong Centre for Emerging Infectious Diseases, Geelong, Victoria Australia; 2 Deakin University, School of Medicine, Geelong, Victoria Australia; 3 WHO Collaborating Centre for Reference and Research on Influenza, the Peter Doherty Institute for Infection and Immunity, Melbourne, Victoria Australia; 4 Centre for Integrative Ecology, School of Life and Environmental Sciences, Deakin University, Geelong, Victoria Australia; 5 Barwon Health, University Hospital Geelong, Geelong, Victoria Australia

**Keywords:** Ecological epidemiology, Viral infection

## Abstract

We evaluated the presence of coronaviruses by PCR in 918 Australian wild bird samples collected during 2016–17. Coronaviruses were detected in 141 samples (15.3%) from species of ducks, shorebirds and herons and from multiple sampling locations. Sequencing of selected positive samples found mainly gammacoronaviruses, but also some deltacoronaviruses. The detection rate of coronaviruses was improved by using multiple PCR assays, as no single assay could detect all coronavirus positive samples. Sequencing of the relatively conserved Orf1 PCR amplicons found that Australian duck gammacoronaviruses were similar to duck gammacoronaviruses around the world. Some sequenced shorebird gammacoronaviruses belonged to Charadriiformes lineages, but others were more closely related to duck gammacoronaviruses. Australian duck and heron deltacoronaviruses belonged to lineages with other duck and heron deltacoronaviruses, but were almost 20% different in nucleotide sequence to other deltacoronavirus sequences available. Deltacoronavirus sequences from shorebirds formed a lineage with a deltacoronavirus from a ruddy turnstone detected in the United States. Given that Australian duck gammacoronaviruses are highly similar to those found in other regions, and Australian ducks rarely come into contact with migratory Palearctic duck species, we hypothesise that migratory shorebirds are the important vector for moving wild bird coronaviruses into and out of Australia.

## Introduction

Coronaviruses (CoV) are the causative agents of significant diseases resulting in substantial impact on human and animal health. Both severe acute respiratory syndrome coronavirus (SARS-CoV) and Middle East respiratory syndrome coronavirus (MERS-CoV) have caused a significant burden on human health, including a number of deaths, and have had significant socioeconomic impacts on the countries in which people were infected^1–3^. Coronavirus infections of livestock, such as porcine epidemic diarrhoea virus (PEDV) and more recently porcine deltacoronavirus (PDCoV) in pigs and infectious bronchitis virus (IBV) and turkey coronavirus in poultry, have significant impacts on animal health and cause considerable economic costs to producers^4–7^.

Interspecies spill-over of coronaviruses into new hosts occurs frequently, with SARS-CoV and MERS-CoV being the most notable examples of spill-over into humans^8–10^. Bovine coronavirus, canine respiratory coronavirus, dromedary camel coronavirus and even human coronavirus OC43 all potentially come from the same common ancestor, illustrating substantive host flexibility^11–13^. SARS-CoV likely originated in bats while PDCoV interestingly is likely to have originated in birds^6,8,9^. Consequently, there is significant interest in assessing wild animals for CoV’s.

Wild birds are ubiquitous and highly mobile potential hosts capable of moving viruses over large distances and across geographical and political borders. Wild birds have been implicated in the spread of highly pathogenic H5Nx avian influenza viruses^14^ and bird migration patterns describe phylogenetic patterns in the matrix gene of low pathogenic influenza virus^15^. Coronaviruses have been detected in a range of species of wild birds on all continents except for Australia and Antarctica^16–21^. It is likely that CoV’s are present in wild birds on all continents. However, a recent survey in Australia of 409 birds failed to detect coronaviruses^22^.

Studies in which wild bird coronaviruses have been successfully detected, have commonly sampled aquatic bird species such as Anseriformes (ducks, geese and swans) and Charadriiformes (gulls, migratory shorebirds and waders)^16,18,20,23,24^. This prompted our study to look for coronaviruses in Australian birds that notably belong to these two orders.

Given that the genetic diversity of CoV’s in Australian wild birds was unknown it was decided to use a combination of PCR assays to maximise the probability of detecting positive individuals. Coronavirus positive samples were genetically sequenced and phylogenetic analysis performed to compare these sequences to coronavirus sequences from previous surveys of wild and domestic birds across the globe.

## Results

### PCR assay comparison

The 5′ UTR detected 66/912 or 7.2% (95% CI: 5.6–8.9%) of samples positive for coronavirus, the nested PCR 100/916 samples or 10.9% (95% CI: 8.9–13.0%) and the modified pancoronavirus PCR 109/915 samples or 11.9% (95% CI: 9.8–14%). By combining the results from the three PCRs, 141/918 or 15.3% (95% CI: 13–17.7%) of all samples tested positive for coronaviruses in at least one PCR assay. Nine hundred and eight samples were tested with all three PCRs. Ten samples had insufficient RNA to allow testing with all three assays. Nine of these samples were negative in the assays with which they were tested. One (Ruddy turnstone CoV-9614–2016/11/19-CM/TAS) was positive in the nested PCR, negative in the 5′ UTR PCR but could not be tested with the modified PCR. These ten samples were excluded from the PCR assay comparison.

When compared to the combined results, the sensitivities for the UTR, nested and modified PCRs were 46.8%, 70.9% and 77.3% respectively. McNemar’s test revealed no statistical difference in the coronavirus detection abilities of the modified and nested PCRs (χ^2^ = 2.38, p = 0.12) but a statistical difference was detected between the results of the UTR PCR and the nested PCR (χ^2^ = 13.96, p = 0.0002) and the UTR and modified PCR (χ^2^ = 21.25, p = 4 × 10^−6^).

From the subset of 66 samples where the Orf1/polymerase PCR amplicon was sequenced and genus of coronavirus identified (excluding the one positive pooled sample which contained both a gamma-CoV and delta-CoV (see later)), the 5′ UTR PCR did not detect any delta-CoV’s and only 50% of the gamma-CoV’s (27/54). The nested PCR detected 75% (9/12) of the delta-CoV’s and 85% (46/54) of the gamma-CoV’s. The modified PCR detected 75% (9/12) of the delta-CoV’s and 87% (47/54) of gamma-CoV’s. No PCR was able to detect all positive samples. The 5′ UTR PCR, despite having the lowest sensitivity, detected 19 samples which were declared negative by the other two PCRs. Similarly, both the nested and modified PCRs detected 3 delta-CoV’s the other missed, despite targeting exactly the same region of the CoV genome.

Coronavirus positive samples produced a peak in the melt curve analysis of the modified polymerase real-time PCR with delta-CoV positive samples producing a peak between 80.6 °C and 81.8 °C and gamma-CoV positive samples producing a peak between 80.4 °C and 85.6 °C. No obvious pattern between melt curve peak temperature (T_M_) or shape was apparent, which could be used to determine the genus of the coronavirus. Samples which produced a visible band of the expected size on the agarose gel also produced a strong peak on the melt curve. However, some negative samples produced small non-specific peaks in the melt curve within, and slightly above, the temperature range in which the positive samples produced peaks. These were small and readily differentiated from strong positive samples. However, without running a gel, these peaks would make it difficult to determine if a sample was weak positive or a non-specific reactor. When present, these non-specific peaks were associated with a ~300 bp band on the agarose gel that was readily differentiated from the target band of size 600 bp. During optimisation of this PCR on known negative and known positive samples, the non-specific band disappeared with forward and reverse primer concentrations of 0.5 µM as opposed to the 1 µM used in this study. This, however, had the impact of increasing the C_T_ of positive samples by about 3 (an approximately 8-fold decrease in PCR amplification) and it was decided to go for increased PCR sensitivity over the removal of the small non-specific product as all positive samples could be confirmed by gel analysis.

### Detection of coronaviruses in Australian wild birds

Coronaviruses were detected in 141 of the 918 samples tested (15.3%). Positive samples were found in eight out of the 15 bird species tested (Table 1). Negative species had very low sample sizes (<30 individuals). Positive species included 4 species of ducks (Order Anseriformes): Pacific black duck (18/48), grey teal (19/63), radjah shelduck (1/3) and Australian wood duck (1/16); three shorebirds (Order Charadriiformes): curlew sandpiper (8/34), red-necked stint (42/534) and ruddy turnstone (47/157); and one species of heron (Order Pelecaniformes): pied heron (5/7). The coronavirus sample prevalence in Anseriformes was 26.7%, 13.3% in the Charadriiformes and 71.4% in the Pelecaniformes. Coronaviruses were only found in apparently healthy birds with none of the dead birds positive for CoV. Coronavirus positive birds were found in all states where sampling was conducted. Details of positive samples are presented in Supplementary Table S1.

**Table 1:** The bird species sampled in this study and the proportion of coronavirus positive samples.

Avian Order/Family	Species	Common Name	coronavirus + ve/Total	Coronavirus Genus
*Anseriformes, Anatidae*	*Anas gracilis*	Grey teal	19/63	Gamma (11)
	*Anas superciliosa*	Pacific black duck	18/48	Gamma (18), Delta (1)*
	*Tadorna radjah*	Radjah shelduck	1/3	Gamma (1)
	*Chenonetta jubata*	Australian wood duck	1/16	
	*Tadorna tadornoides*	Australian shelduck	0/5	
	*Anas castanea*	Chestnut teal	0/11	
*Pelecaniformes, Ardeidae*	*Ardea picata*	Pied heron	5/7	Delta (5)
*Charadriiformes, Scolopacidae*	*Caladris ferruginea*	Curlew sandpiper	8/34	Gamma (3), Delta (1)
	*Caladris ruficolis*	Red-necked stint	42/534	Gamma (9), Delta (2)
	*Calidris acuminata*	Sharp-tailed sandpiper	0/3	
	*Arenaria interpres*	Ruddy turnstone	47/157	Gamma (20), Delta (5)
*Procellariiformes*	*Ardenna tenuirostris*	Short-tailed shearwater	0/30	
*Acciptriformes*	*Milvus migrans*	Black kite	0/1	
*Columbiformes*	*Streptopelia chinensis*	Spotted turtle dove	0/1	
*Passeriformes*	*Corvus mellori*	Little Raven	0/4	

*One pooled sample contained both a gammacoronavirus and deltacoronavirus.

Of the 141 positive samples, 67 were sequenced to identify the genus of CoV present. Samples for sequencing were a subset of samples which were positive in either the nested and/or modified PCRs targeting the polymerase gene. Approximately 25–50% of positive samples per species were sequenced, except in the species for which very few samples were tested (e.g. the pied herons and the radjah shelduck). In these species with small sample sizes, all positive samples were sequenced. Gamma-CoV’s (n = 54) were more frequently identified than delta-CoV’s (n = 13). The pooled sample from juvenile Pacific black ducks contained both a gamma- and a delta-CoV. This was the only sample in which two coronaviruses were detected. In the order Anseriformes, 22 gamma-CoV’s (grey teals (11), Pacific black ducks (10) and radjah shelduck (1)) and one delta-CoV (Pacific black duck) were identified. In the order Charadriiformes, 32 gamma-CoV’s (curlew sandpipers (3), red-necked stints (9), ruddy turnstones (20)) and 8 delta-CoV’s (curlew sandpiper (1), red-necked stints (2), ruddy turnstones (5)) were identified. In order Pelecaniformes, 5 delta-CoV’s were sequenced (pied herons (5)) and no gamma-CoV’s were identified.

### Phylogenetic analysis of gammacoronaviruses

**Figure 1:**
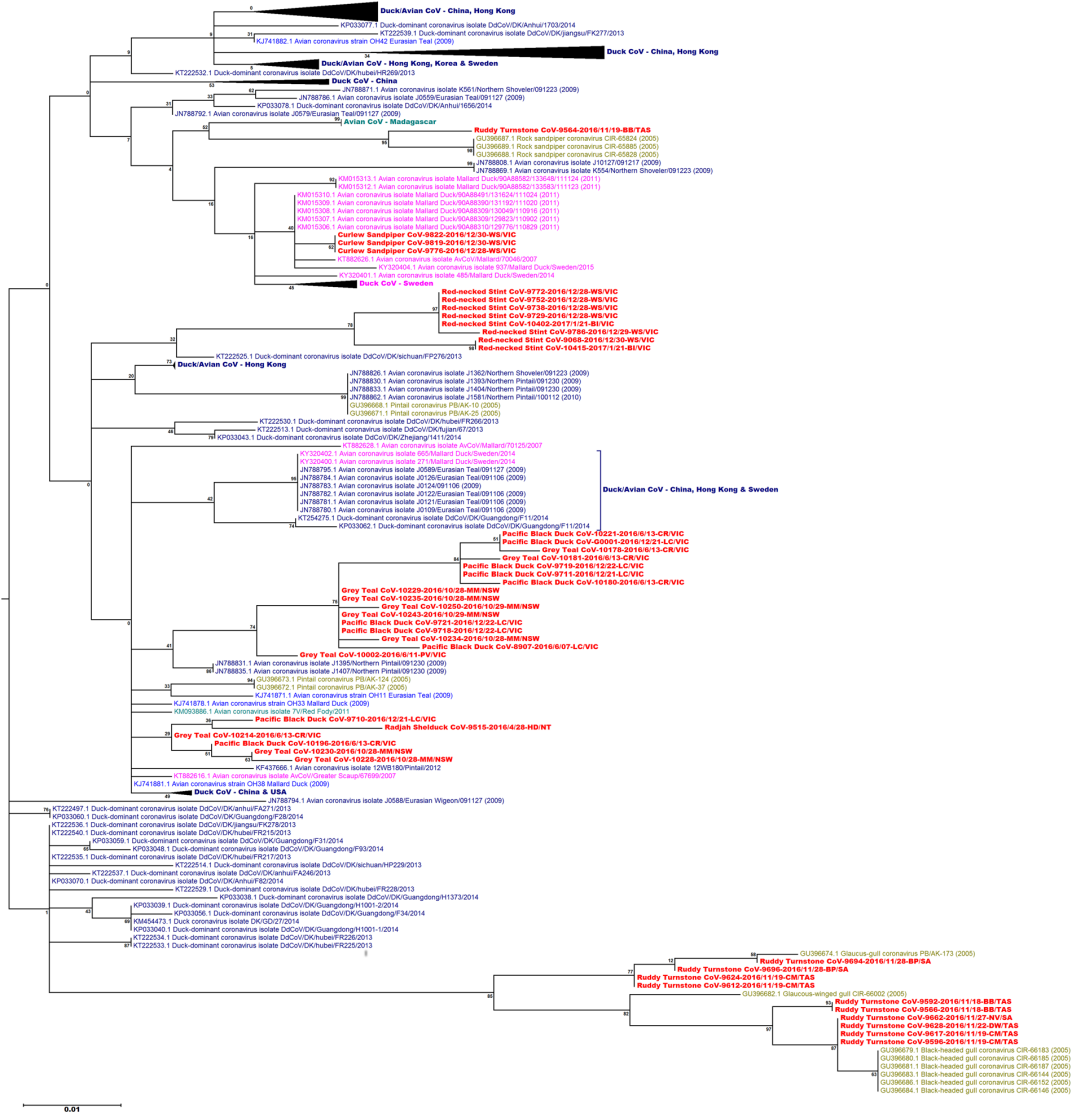
Maximum likelihood phylogenetic tree of the 277 nt fragment of the polymerase gene of gammacoronaviruses from wild birds including the sequences obtained in this study. Bootstrap confidence of each branch was calculated from 1000 replicates. The region where each sequence was obtained is indicated with colour. Australia (red), Madagascar (teal), China, Hong Kong and Korea (dark blue), Bering Strait (green), United States of America (light blue) and Sweden (pink). The sampling location and state of each Australian sample is identified with a two letter code and the state: New South Wales (NSW): MM-Moulamein; Northern Territory (NT): HD-Humpty Doo; South Australia (SA): BP-Boatswain Point, NV-Nene Valley; Tasmania (TAS): BB-Borges Bay, King Island, CM-Central Manuka, King Island, DW-Dripping Wells, King Island, Tasmania; Victoria (VIC): BI-Barrallier Island, CR-Carlisle River, LC-Lake Connewarre, PV-Paynesville, WS-Werribee South. Some branches have been collapsed if all sequences came from the same region. The complete tree is shown in Supplementary Figure S1.

Gammacoronavirus sequences from this study were more related to gamma-CoV’s from ducks and wild birds (Fig. 1) than to IBV-like gamma-CoV’s including IBVs from Australia (Supplementary Figure S1). All Australian duck gamma-CoV’s grouped on two branches within a predominantly duck lineage of gamma-CoV’s. Bootstrap support for the branches containing the Australian sequences was less than 75%. There was no apparent host duck species, temporal or geographical pattern to the Australian duck gamma-CoV’s distribution within the phylogenetic tree.

Gamma-CoV’s from shorebirds grouped on branches with other shorebird gamma-CoV’s sequences obtained in this study or from the “*Beringia*” study of wild birds around the Bering Strait^20^. With the exception of curlew sandpiper gamma-CoV’s, these branches had 75% or greater bootstrap support in the phylogenetic analysis. There was sequence diversity in the gamma-CoV sequences in all shorebird species except the curlew sandpipers. The majority of the ruddy turnstone gamma-CoV’s grouped with gamma-CoV’s from gulls sampled in 2005 during the Beringia study. One ruddy turnstone gamma-CoV grouped with gamma-CoV’s from rock sandpipers from the Beringia study. Red-necked stint gamma-CoV’s sequences grouped on their own branch. The three curlew sandpiper gamma-CoV sequences were identical, and were most similar to CoV sequences from ducks sampled in Sweden during 2011^25^.

**Figure 2:**
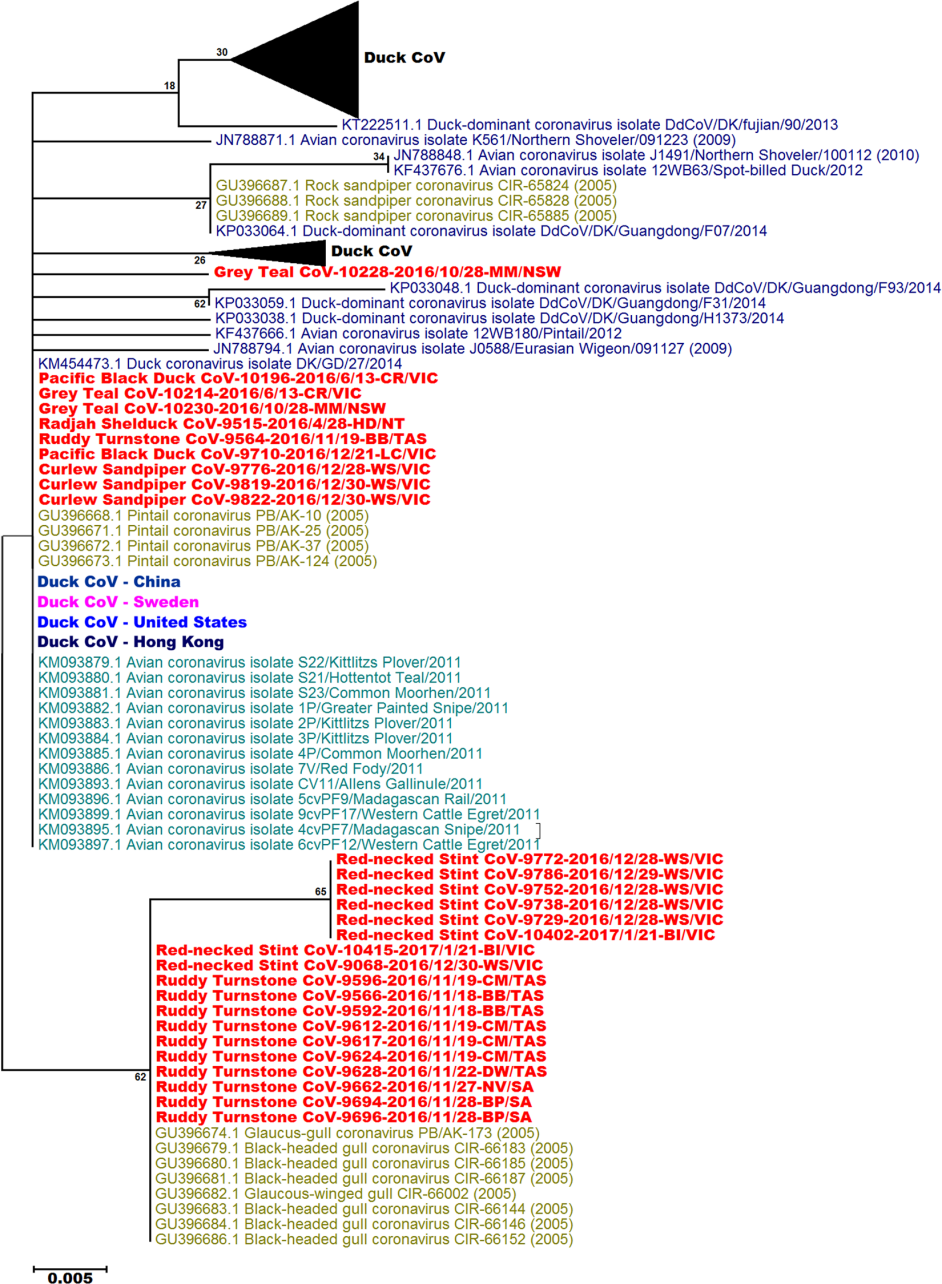
Maximum likelihood phylogenetic tree of the 92 amino acid sequence of the gammacoronaviruses from wild birds. Bootstrap confidence of each branch was calculated from 500 replicates. Some branches have been collapsed for clarity. The region where each sequence was obtained is indicated with colour. Australia (red), Madagascar (teal), China, Hong Kong and Korea (dark blue), Bering Strait (green), United States of America (light blue) and Sweden (pink). The sampling location and state of each Australian sample is identified with a two letter code and the state: New South Wales (NSW): MM-Moulamein; Northern Territory (NT): HD-Humpty Doo; South Australia (SA): BP-Boatswain Point, NV-Nene Valley; Tasmania (TAS): BB-Borges Bay, King Island, CM-Central Manuka, King Island, DW-Dripping Wells, King Island, Tasmania; Victoria (VIC): BI-Barrallier Island, CR-Carlisle River, LC-Lake Connewarre, PV-Paynesville, WS-Werribee South. Some branches have been collapsed if all sequences came from the same region. The complete tree is shown in Supplementary Figure S2.

The amino acid ML tree of the gamma-CoV separated the majority of the shorebird gamma-CoV’s and gull gamma-CoV’s from the duck-CoV’s (Fig. 2, Supplementary Figure S2). However the three curlew sandpiper CoV’s and one ruddy turnstone CoV (Ruddy Turnstone CoV-9564-2016/11/19-BB/TAS) were identical in amino acid sequence to several Australian duck gamma-CoV’s, as well as many domestic and wild duck gamma-CoV sequences from Asia, Africa, Europe and North America.

### Phylogenetic analysis of deltacoronaviruses

**Figure 3:**
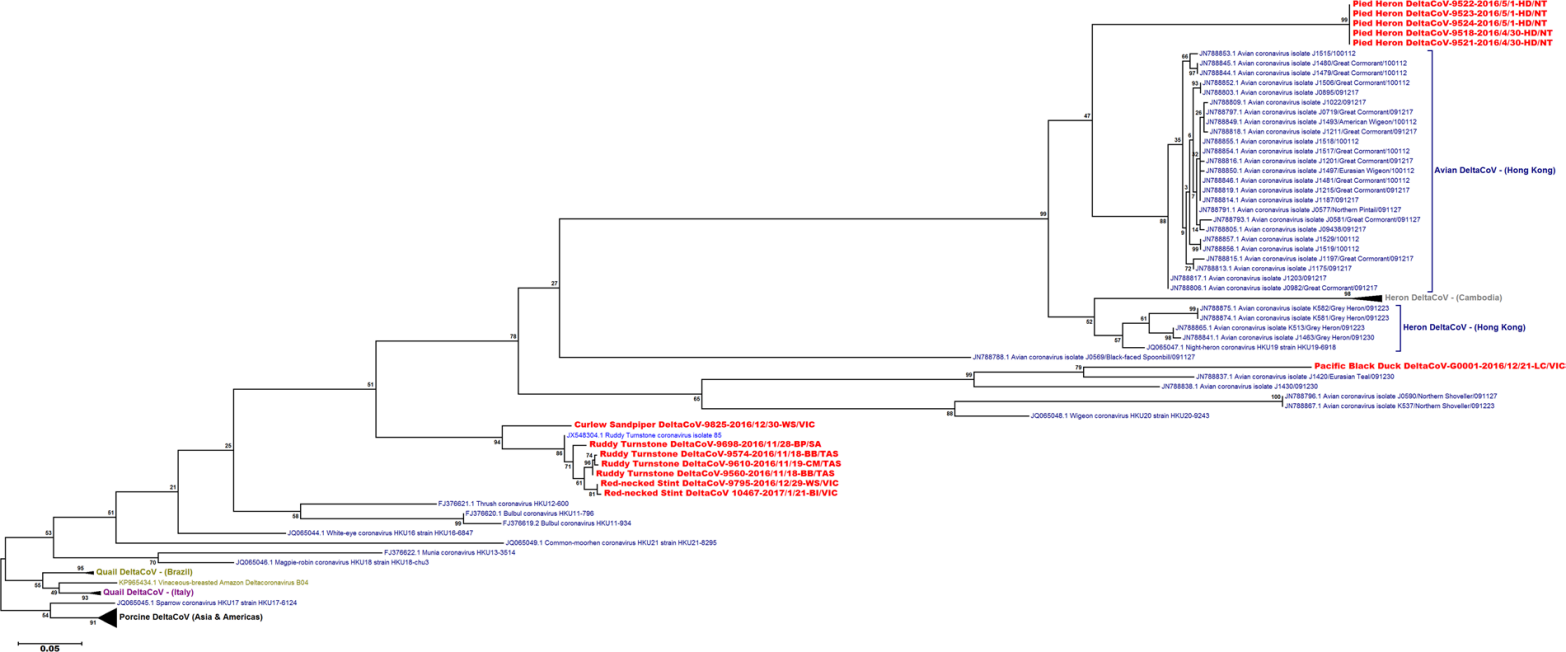
Maximum likelihood phylogenetic tree of the 311 nt fragment of the polymerase gene of deltacoronaviruses from wild birds, including the sequences obtained in this study. Bootstrap confidence of each branch was calculated from 1000 replicates. The region where each sequence was obtained is indicated with colour. Australia (red), Madagascar (teal), China, Hong Kong and Korea (dark blue), Bering Strait region (green), United States of America (light blue) and Sweden (pink). The sampling location and state of each Australian sample is identified with a two letter code and the state: New South Wales (NSW): MM-Moulamein; Northern Territory (NT): HD-Humpty Doo; South Australia (SA): BP-Boatswain Point, NV-Nene Valley; Tasmania (TAS): BB-Borges Bay, King Island, CM-Central Manuka, King Island, DW-Dripping Wells, King Island, Tasmania; Victoria (VIC): BI-Barrallier Island, CR-Carlisle River, LC-Lake Connewarre, PV-Paynesville, WS-Werribee South. Some branches have been collapsed if all sequences came from the same region. The complete tree is shown in Supplementary Figure S3.

Deltacoronavirus sequences from this study were spread across three lineages in the ML phylogenetic analysis (Fig. 3, Supplementary Figure S3). Delta-CoV’s from ruddy turnstones, red-necked stints and curlew sandpipers (all from order Charadriiformes) formed a lineage with a delta-CoV sequence from a ruddy turnstone sampled in the United States. The single Australian duck delta-CoV belonged to a duck lineage with duck delta-CoV’s detected in Hong Kong but was 18.6% different to its nearest relative. The pied heron delta-CoV sequences were all identical, and belonged to a lineage which included delta-CoV’s from species from avian orders Pelecaniformes (Herons), Suliformes (Cormorants) and Anseriformes (Ducks) from Hong Kong and Cambodia. The pied heron delta-CoV was 17.7% different to the next most similar sequence. The genetic diversity in the Charadriiformes delta-CoV lineage was low (1.9–3.2% nucleotide differences) compared to the other lineages (duck lineage 18.6–30.9%; heron/cormorant lineage 17.7–22.8%). The inter-lineage genetic variation was high between wild bird delta-CoV’s. For example the Australian duck delta-CoV was 31.8–34.4% different to the delta-CoV’s within the Heron/Cormorant lineage and 29.6–31.8% different to sequences within the Chardriiforme lineage.

## Discussion

This study is the first to demonstrate the presence of coronaviruses in wild birds in Australia. Coronaviruses were distributed across multiple species and geographical locations, with an average coronavirus positive prevalence of 15.3%. In bird orders with over 50 individuals sampled, coronaviruses were most frequently detected in Anseriformes (26.7%) followed by Charadriiformes (13.3%). Gammacoronaviruses were found in 100% of sequenced samples from ducks, while duck deltacoronaviruses were much less common (in only one sample). Shorebirds were positive for gamma-CoV’s most commonly (80% of sequenced samples), but delta-CoV’s were also present in these species (20% of sequenced samples). Herons were positive for delta-CoV’s exclusively; however, with the caveat that only 7 samples were included in this study. While the patterns of predominantly gammacoronaviruses in ducks and deltacoronaviruses in herons have been described in previous surveys^16,19,20,23^, the detection of both gamma- and delta-CoV’s in the same populations of Charadriiformes at the same time is unique to this study.

DNA sequencing of 67 of the 141 PCR positive samples confirmed that the PCRs were in fact amplifying coronavirus cDNA, although it was evident that no single PCR assay could detect all coronavirus positive samples. The use of multiple PCR assays to screen wild bird samples increased the overall sensitivity for detecting coronaviruses. Compared to the 5′ UTR PCR, nested and modified PCRs alone, the detection rate of coronaviruses increased 2.1, 1.4 and 1.3 fold respectively when all PCRs were used together. Had only one assay been used in this study, a number of coronaviruses in individual species would not have been detected. For example, only the modified PCR detected delta-CoV’s in the curlew sandpipers and the red-necked stints, while the nested PCR was able to detect more delta-CoV’s in ruddy turnstones and pied herons. None of the PCRs described in this study detected the deltacoronavirus in the pooled duck sample. Amplicon sequencing from this sample found that the PCRs were preferentially amplifying the gammacoronavirus present. However next generation sequencing data from this sample (Vibin *et al*., 2018 (submitted)) found deltacoronavirus sequences were more abundant than those of the gammacoronavirus. Therefore the current pan-coronavirus PCRs may favour detection of gammacoronaviruses over deltacoronaviruses and this may explain why studies to date have found many more gammacoronaviruses in wild birds. It is possible that there are many more genetically diverse coronaviruses circulating in wild birds which are currently outside the detection abilities of the current published pancoronavirus PCRs, and it will be interesting to see how our understanding of coronavirus diversity improves as the use of non-targeted next generation sequencing techniques becomes more common.

The small fragment of the coronavirus genome sequenced in this study is one of the most conserved regions of the viral polymerase gene^24^. It is the most sequenced genomic region of coronaviruses from wild bird hosts, with many studies using PCRs targeting this region to detect avian coronaviruses^16,18,24,26^. The fragment analysed in this study represents about 1% of the coronavirus genome, and therefore any interpretation of relationships in the phylogenetic tree should be made cautiously. With these caveats in mind, no temporal or spatial patterns in coronavirus sequences was observed in either the gamma- or deltacoronavirus phylogenetic analyses. However, some clustering by host species/order was observed, and this was strongest in the deltacoronaviruses, similar to the situation observed by Chu, *et al*.^16^ in Hong Kong and Cambodia who observed similar host species lineages. There was a Charadriiformes delta-CoV lineage observed which was strongly supported by the bootstrap analysis. All seven Australian shorebird delta-CoV’s from three different host species clustered with the single previously characterised shorebird delta-CoV from a ruddy turnstone sampled on the East Coast of the United States^21^. The one Australian duck deltacoronavirus belonged to a duck lineage of deltacoronaviruses, but itself was 18.6% different to its closest relative sequence from a Eurasian teal in Hong Kong^16^. Similarly, the Australian heron deltacoronaviruses were within the lineage containing other herons but were genetically different to heron sequences seen elsewhere.

There were Charadriiformes lineages within the gammacoronavirus phylogenetic tree, and the majority of the Australian Charadriiform gamma-CoV’s grouped on branches with other gamma-CoV’s from host species within this order. However some Charadriiform gammacoronaviruses were highly similar in nucleotide sequence to gammacoronaviruses from ducks, e.g. the curlew sandpiper gammacoronaviruses contained a single nucleotide difference to mallard gammacoronavirus sequences identified in Sweden^25^. Even within the Charadriiformes lineages, the degree of genetic difference to duck sequences was much less than that seen in the deltacoronavirus phylogeny. The phylogeny based on the amino acid sequence of the gammacoronaviruses separated the majority of the duck and Charadriiform gammacoronaviruses into separate branches, however both the curlew sandpiper gammacoronaviruses and a single ruddy turnstone gamma-CoV were identical in amino acid homology to duck Australian duck gamma-CoV’s as well as duck gamma-CoV’s identified in other investigations^16,18,20,25,27^. These results again suggest that migratory Charadriiformes may share gammacoronaviruses with other avian species^16,18^ and are therefore important vectors and reservoir of coronaviruses. Hughes, *et al*.^23^ and Chu, *et al*.^16^ identified similar gammacoronavirus sequences between Charadriiformes and Anseriformes, while Lima, *et al*.^18^ found similar gammacoronaviruses in Charadriiformes and Gruiformes in Madagascar. Interestingly, Hughes, *et al*.^23^ identified their similar sequences by a PCR targeting the 3′ UTR, while Lima, *et al*.^18^ identified similar sequences with the same nested PCR used in this investigation^16^.

Regular spill-over of coronaviruses from Charadriiformes to Anseriformes could explain why many Australian duck gammacoronaviruses were similar to the duck gammacoronaviruses from Asia and more distant regions. Australian duck species rarely come into direct contact with Palearctic waterfowl^28^. Some degree of geographical gammacoronavirus lineage formation might therefore have been expected, if this was the only means of transmission of duck gamma-CoV’s, due to the high rate of mutation and recombination observed in other avian gamma-CoV’s^29,30^. However, such isolation was not observed, which suggests that movement of coronaviruses between these populations of ducks occurs frequently. Given that millions of Charadriiformes migrate to Australia to spend their non-breeding season in Australia^31^, Australian ducks would be much more likely to come into direct contact with Palearctic Charadriiformes than Anseriformes. It is also likely that there are many opportunities for individuals within the migrating shorebird populations to acquire and transmit coronaviruses to/from ducks as they move north-south, as coronaviruses are prevalent in both Charadriiformes and Anseriformes along the East Asian-Australasian Flyway^16,20,32^ and these species forage and breed in similar habitats^33^.

This study further highlights the need for greater genetic characterisation of wild bird coronaviruses from all regions of the world to better understand the dispersal and host spill-over dynamics of these important pathogens. Further work to obtain more sequence data from the Australian wild bird coronaviruses is currently underway.

## Materials and Methods

### Sample collection and storage

Samples tested in this investigation (918) originated from two sources, avian influenza A surveillance by Deakin University (912) and a few dead birds submitted through the Wildlife Health Surveillance Victoria program (6) at the University of Melbourne’s Faculty of Veterinary and Agricultural Science, Werribee, Victoria. Most samples were taken from birds within avian families Anatidae (Anatinae and Tadorninae; Order Anseriformes – 146 samples), Scolopacidae (Order Charadriiformes - 728 samples), Procellariidae (Order Procellariiformes – 30 samples) and Ardeidae (Order Pelecaniformes – 7 samples). Birds were sampled between April 2016 and January 2017 at multiple locations in south eastern Australia and the Northern Territory. Birds were caught either by hand, cannon netting or baited walk-in traps^34^. Some duck samples were obtained from hunter-killed animals. All samples were taken from single individuals and consisted of a combined cloacal and oropharyngeal swab, except for one sample (Pacific Black Duck CoV-G0001–2016/12/21-LC/VIC), which was a pool of faeces collected from a group of 6 juvenile Pacific black ducks trapped together. No birds sampled as part of the influenza surveillance were showing any clinical signs of disease. Capture and sampling of birds were carried out in accordance with all relevant guidelines and regulations and under animal ethics permits B37-2013 and B43-2016 issued by Deakin University Animal Ethics Committee.

Oropharyngeal and cloacal swabs from birds belonging to other families (Accipitridae (Order Accipitriformes) Columbidae (Order Columbiformes), Corvidae (Order Passeriformes)) were collected from six dead birds. Post mortem revealed the cause of death in the four little ravens (*Corvus mellori*) and one black kite (*Milvus migrans*) as physical trauma. Only the spotted turtle dove (*Streptopelia chinensis*) showed clinical signs of disease (diarrhoea and depression) and had been euthanised by a veterinarian (no post mortem was performed on this bird).

Oropharyngeal and cloacal swabs were pooled in viral transport media (brain heart infusion [BHI] broth based medium [Oxoid] with 0.3 mg/ml penicillin, 5 mg/ml streptomycin, 0.1 mg/ml gentamicin, and 2.5 g/ml amphotericin B). Swabs were placed in viral transport media at the collection sites, transported at 4 °C to the laboratory and stored at −80 °C until the nucleic acid extraction was performed.

### Extraction of RNA

Total viral nucleic acids from captured wild bird samples was extracted using the Nucleomag Vet Viral DNA/RNA isolation Kit (Scientifix, Australia) and a Kingfisher™ Flex extraction robot (Life Technologies) as per the manufacturer’s instructions, with an elution volume of 50 ul. RNA from the six samples collected from the dead birds was extracted using the Viral RNA mini-prep kit (Qiagen, Australia) as per the manufacturer’s instructions. Extracted RNA was stored at −80 °C. RNA from infectious bronchitis virus (IBV) N1/62 cultured in specified pathogen free chicken eggs (allantoic fluid, passage 7) was extracted using the Viral RNA mini-prep kit and used as a PCR positive control in this investigation. The RNA of each sample was then used in three separate PCR assays as described below.

### 5′ Untranslated region (UTR) coronavirus real-time PCR

A real-time TaqMan PCR, originally developed to detect IBV in commercial poultry^35^, and later used to screen wild birds for the presence of coronaviruses^17^, was adapted into a real-time SYBR Green PCR assay using the Power SYBR Green RNA-to-C_T_ 1 step kit (Applied Biosystems, California, USA). The PCR amplified a 142 bp product from the 5′ UTR of gammacoronaviruses using primers IBV5′GU391 (5′-GCT TTT GAG CCT AGC GTT-3′) and IBV5′GL533 (5′-GCC ATG TTG TCA CTG TCT ATT G-3′). 2 µl of sample RNA was added to 1 × Power SYBR Green RT-PCR Mix, 1 uM of each primer, 1 × RT-Enzyme Mix and RNase free water to make a total reaction volume of 10 µl. The reaction was conducted in a QuantStudio™ Flex 6 real-time thermal cycler at 48 °C for 30 min, 95 °C for 10 min, 40 cycles of 95 °C for 15 sec, 60 °C for 10 sec, 72 °C for 20 sec and a final 72 °C step for 3 min. A melt curve analysis was performed immediately post PCR with the reaction conditions of 95 °C for 15 sec, then 60 °C for 1 min followed by a continuous temperature ramp between 60 °C and 95 °C increasing at 0.05 °C/sec. The IBV positive control produced a single peak melt curve with a peak melt temperature (T_M_) of 80.25 ± 0.3 °C. If a sample produced a melt curve with a T_M_ within 0.3 °C of the IBV positive control, 5 µl was then run on a 2% agarose gel. A sample was declared positive if it produced a single peak melt curve consistent with the IBV positive control and produced a 140 bp product on the agarose gel.

### Pancoronavirus polymerase nested PCR

A nested pancoronavirus PCR^36^ originally developed to detect coronaviruses in bats and later modified and used to detect gammacoronaviruses and deltacoronaviruses in wild birds^16^ was used to detect coronaviruses in this investigation. The primers used in the initial PCR were: Chu-RdRp-N1-F (5′-GGK TGG GAY TAY CCK AAR TG-3′) and Chu-RdRp-N1-R (5′-TGY TGT SWR CAR AAY TCR TG-3′) and amplified a 602 bp product from the polymerase gene of coronaviruses. cDNA synthesis was performed using the High-Capacity cDNA Reverse Transcription Kit (Thermo Fisher, Waltham, MA, USA) using 1 × random hexamers as per the manufacturer’s protocol. The initial PCR reaction was made with 2 µl of cDNA, 1 × AmpliTaq Gold 360 PCR Mix (Applied Biosystems, California USA), 0.5 µM of each primer, 3 mM MgCl_2_ and nuclease free water in a total volume of 10 µl. The nested PCR used primers Chu-RdRp-N2-F (5′-GGT TGG GAC TAT CCT AAG TGT GA-3′) and Chu-RdRp-N2-R (5′-CCA TCA TCA GAT AGA ATC ATC AT-3′) and amplified a 440 bp product within the first PCR. The PCR reaction mixture was made with 1 µl of the first PCR reaction, 1 × AmpliTaq Gold 360 PCR Mix, 0.5 µM of each primer, 3 mM MgCl_2_ and RNase free water in a total volume of 10 µl. PCR reactions were conducted in a thermal cycler at 95 °C for 10 min, then 40 cycles of 95 °C for 30 sec, 48 °C for 30 sec, 72 °C for 50 sec followed by a final step of 72 °C for 3 min. 5 µl of each PCR was then run on a 1.6% agarose gel. A sample was declared positive if a band of the expected size was seen on the gel from either PCR.

### Modified pancoronavirus polymerase real-time PCR

A non-nested touchdown real-time PCR was developed and evaluated against the nested pancoronavirus PCR to see if it could be used as a replacement for the nested assay in future studies. Primers were designed incorporating all current coronavirus polymerase gene sequences and looking for the most conserved regions. Primer candidates were then evaluated in PerlPrimer^37^. The selected primers, AC-CoV-F (5′-GGT TGG GAT TAT CCW AAR TGT G-3′) and AC-CoV-R (5′-TGY TGT GAR CAA AAY TCR TG-3′), were less degenerate than Chu-RdRp-N1-F and Chu-RdRp-N1-R described above and targeted the same conserved sites in the polymerase gene of coronaviruses producing a PCR product of 602 bp. cDNA synthesis was performed using the High-Capacity cDNA Reverse Transcription Kit (Thermo Fisher, Waltham, MA, USA) as per the manufacturer’s instructions but using 100 pM of the reverse primer AC-CoV-R in place of the 1 × random hexamers. The PCR reaction was performed using 2 µl of cDNA, 1 × AmpliTaq Gold 360 PCR Mix, 1 µM of each primer, 3 mM MgCl_2_, 2 µM Syto 9 (Thermo Fisher, Waltham, MA, USA) and molecular grade water to a final volume of 10 µl. The reaction was conducted in a QuantStudio™ Flex 6 real-time thermal cycler at 95 °C for 10 min, 45 cycles of 95 °C for 30 sec, annealing temperature for 30 sec, 72 °C for 45 sec and a final 72 °C step for 3 min. The touchdown annealing temperature started at 60 °C for 3 cycles, then decreased by 2 °C every 3 cycles until 48 °C which was used for the final 30 reaction cycles.

A melt curve analysis was performed immediately post PCR with reactions subjected to 95 °C for 15 sec, then 60 °C for 1 min followed by a continuous temperature ramp between 60 °C and 95 °C increasing at 0.05 °C/sec. 5 µl of the PCR product was then run on a 1.6% agarose gel and a sample was declared positive if a band of the expected size was seen.

### PCR product sequencing and phylogenetic analysis

A selection of 67 PCR products from coronavirus Orf1/polymerase PCR positive samples were gel purified using the 2% Size Select E-Gel System (Thermo Fisher, Waltham, MA, USA) and sequenced using the Big Dye Terminator Cycle v3.1 on a Hitachi 3500xl genetic analyser (Applied Biosystems, Foster City, California, USA). As a result of PCR product sequencing conducted during the preliminary PCR testing and optimisation, the pooled duck sample (Pacific Black Duck CoV-G0001-2016/12/21-LC/VIC) was found to contain both a gamma-CoV and a delta-CoV. The gamma-CoV sequence used in this analysis was obtained as described above, however the delta-CoV sequence was obtained from the analysis performed by Vibin, *et al*.^38^ (submitted). All sequences generated in this study have been deposited in Genbank under accession numbers MG764091 to MG764157 and MH090080 and MH090081.

DNA sequences were aligned in MEGA 7^39^. Each sequence was then trimmed to where both forward and reverse reads 100% agreed on the consensus sequence. Consensus sequences between 315 and 515 nt were generated and queried against the Genbank database using a BLASTn query to identify the genus of coronavirus. Ten amplicons which were too short or did not have 100% agreement between the forward and reverse strands were excluded from subsequent multiple sequence alignments and phylogenetic analysis.

A final dataset (57) of 44 gamma-CoV and 13 delta-CoV sequences were aligned with gamma- and delta-CoV sequences available on Genbank using the ClustalW algorithm in MEGA 7. The aligned region belonged to the Orf1/Polymerase gene between nucleotides 14203 to 14761 of the reference avian infectious bronchitis virus sequence (Genbank accession: NC_001451.1). Absence of evidence of recombination was confirmed using the Genetic Algorithm Recombination Detection (GARD) method^40^ of the HyPhy package hosted on the DataMonkey webserver^41^. Codon specific selection pressure was analysed using the single likelihood ancestor counting (SLAC), fixed effects likelihood (FEL), internal fixed effects likelihood (IFEL) and random effects likelihood (REL) methods available on the DataMonkey server as previously described^42^. All analyses were performed on the optimum model as determined by the model selection algorithm of the server, and using Neighbour-Joining phylogenetic trees. A p-value of 0.05 or Bayes factor of 100 were selected for the selection pressure analyses, which indicated evidence for negative selection and no evidence of positive selection (data not shown).

To understand the relationship between the coronavirus sequences identified in this study and those of other studies, a maximum likelihood (ML) phylogenetic analysis of the nucleotide sequences with 1000 bootstrap replicates was performed in MEGA 7. The gammacoronavirus phylogenetic tree was created from 277 nt of sequence using the Tamura-Nei model with 5 discrete gamma distribution categories^43^. The deltacoronavirus phylogenetic tree was created from 311 nt of sequence using the Tamura 3 parameter model^44^ with 5 discrete gamma distribution categories with invariant sites. An amino acid maximum likelihood phylogenetic analysis was performed on the gammacoronavirus sequence (92 amino acids) using the Jones-Taylor-Thornton model with 5 discrete gamma distribution categories and 500 bootstrap replicates. All models were determined to be the best fitting models to the sequence data by MEGA 7.

### Statistical and data analysis

A sample was considered positive for coronavirus if it was positive in at least one of the PCR assays. The agreement between the pancoronavirus PCRs was evaluated using the McNemar’s test with Bonferroni’s correction and a significance value (α) of 0.05^45^.

### Data availability

All sequences generated have been deposited in GenBank under accession numbers MG764091 - MG764157 and MH090080 and MH090081. Other datasets generated or analysed during the current study are available from the corresponding author on reasonable request.

## Electronic supplementary material

Supplementary Information
