# Supplementary material for: Detection and characterisation of coronaviruses in migratory and non-migratory Australian wild birds
Source: Sci Rep. 2018 Apr 13;8:5980. doi: 10.1038/s41598-018-24407-x (PMC5899083; doi:10.1038/s41598-018-24407-x)
Supplement: Supplementary file 1 — Supplementary Information [file 41598_2018_24407_MOESM1_ESM.pdf]

Supplementary information

Detection and characterisation of coronaviruses in migratory and non-migratory Australian wild birds

Anthony Chamings[1,2], Tiffanie M. Nelson[1,2], Jessy Vibin[1,2], Michelle Wille[3], Marcel Klaassen[4] and Soren Alexandersen[1,2,5 a]

[1]Geelong Centre for Emerging Infectious Diseases, Geelong, Victoria, Australia.

[2]Deakin University, School of Medicine, Geelong, Victoria, Australia.

[3]WHO Collaborating Centre for Reference and Research on Influenza, the Peter Doherty Institute for Infection and Immunity, Melbourne, Victoria, Australia.

[4]Centre for Integrative Ecology, School of Life and Environmental Sciences, Deakin University, Geelong, Victoria, Australia.

[5]Barwon Health, University Hospital Geelong, Geelong, Victoria, Australia.

[a]Corresponding author: soren.alexandersen@deakin.edu.au

Supplementary Table S1. Bird species, location and timing of sampling for coronavirus positive birds. Age of bird was identified as Unknown (U), Juvenile (J) or adult (A)

Sample ID	Species	Collection Date	Collection Location	State	Age	5' UTR PCR Result	Nested PCR Result	Modified PCR Result	Coronavirus Genus	Genbank Accession
Curlew sandpiper CoV-9763-2016/12/28-WS/VIC	Curlew sandpiper	28/12/2016	Werribee South	VIC	U	-	-	+		
Curlew sandpiper CoV-9768-2016/12/28-WS/VIC	Curlew sandpiper	28/12/2016	Werribee South	VIC	U	-	+	+		
Curlew sandpiper CoV-9776-2016/12/28-WS/VIC	Curlew sandpiper	28/12/2016	Werribee South	VIC	U	-	+	+	Gamma	MG764091
Curlew sandpiper CoV-9819-2016/12/30-WS/VIC	Curlew sandpiper	30/12/2016	Werribee South	VIC	U	+	-	+	Gamma	MG764092
Curlew sandpiper CoV-9820-2016/12/30-WS/VIC	Curlew sandpiper	30/12/2016	Werribee South	VIC	U	-	+	+		
Curlew sandpiper CoV-9821-2016/12/30-WS/VIC	Curlew sandpiper	30/12/2016	Werribee South	VIC	U	-	-	+		
Curlew sandpiper CoV-9822-2016/12/30-WS/VIC	Curlew sandpiper	30/12/2016	Werribee South	VIC	U	-	+	-	Gamma	MG764093
Curlew sandpiper CoV-9825-2016/12/30-WS/VIC	Curlew sandpiper	30/12/2016	Werribee South	VIC	U	-	-	+	Delta	MG764094
Grey teal CoV-10002-2016/6/11-PV/VIC	Grey teal	11/06/2016	Paynesville	VIC	U	-	+	-	Gamma	MG764095
Grey teal CoV-10178-2016/6/13-CR/VIC	Grey teal	13/06/2016	Carlisle river	VIC	A	+	+	+	Gamma	MG764096
Grey teal CoV-10181-2016/6/13-CR/VIC	Grey teal	13/06/2016	Carlisle river	VIC	A	-	+	+	Gamma	MG764097
Grey teal CoV-10214-2016/6/13-CR/VIC	Grey teal	13/06/2016	Carlisle river	VIC	A	+	+	+	Gamma	MG764098
Grey teal CoV-10215-2016/6/13-CR/VIC	Grey teal	13/06/2016	Carlisle river	VIC	A	+	+	+		
Grey teal CoV-10226-2016/10/28-MM/NSW	Grey teal	28/10/2016	Moulamein	NSW	A	+	+	+		
Grey teal CoV-10227-2016/10/28-MM/NSW	Grey teal	28/10/2016	Moulamein	NSW	A	+	+	+		
Grey teal CoV-10228-2016/10/28-MM/NSW	Grey teal	28/10/2016	Moulamein	NSW	A	-	+	+	Gamma	MG764099
Grey teal CoV-10229-2016/10/28-MM/NSW	Grey teal	28/10/2016	Moulamein	NSW	A	+	+	+	Gamma	MG764100
Grey teal CoV-10230-2016/10/28-MM/NSW	Grey teal	28/10/2016	Moulamein	NSW	A	+	+	+	Gamma	MG764101
Grey teal CoV-10234-2016/10/28-MM/NSW	Grey teal	28/10/2016	Moulamein	NSW	A	+	+	+	Gamma	MG764102
Grey teal CoV-10235-2016/10/28-MM/NSW	Grey teal	28/10/2016	Moulamein	NSW	A	-	+	-	Gamma	MG764103
Grey teal CoV-10237-2016/10/28-MM/NSW	Grey teal	28/10/2016	Moulamein	NSW	A	-	+	+		
Grey teal CoV-10238-2016/10/28-MM/NSW	Grey teal	28/10/2016	Moulamein	NSW	A	-	+	+		
Grey teal CoV-10239-2016/10/28-MM/NSW	Grey teal	28/10/2016	Moulamein	NSW	A	-	+	+		
Grey teal CoV-10240-2016/10/28-MM/NSW	Grey teal	28/10/2016	Moulamein	NSW	A	-	+	+		
Grey teal CoV-10241-2016/10/29-MM/NSW	Grey teal	29/10/2016	Moulamein	NSW	U	-	+	+		

Grey teal CoV-10243-2016/10/29-MM/NSW	Grey teal	29/10/2016	Moulamein	NSW	U	-	+	-	Gamma	MG764104
Grey teal CoV-10250-2016/10/29-MM/NSW	Grey teal	29/10/2016	Moulamein	NSW	U	+	+	+	Gamma	MG764105
Pacific black duck CoV-10180-2016/6/13-CR/VIC	Pacific black duck	13/06/2016	Carlisle river	VIC	A	-	+	+	Gamma	MG764106
Pacific black duck CoV-10196-2016/6/13-CR/VIC	Pacific black duck	13/06/2016	Carlisle river	VIC	A	+	+	+	Gamma	MG764107
Pacific black duck CoV-10221-2016/6/13-CR/VIC	Pacific black duck	13/06/2016	Carlisle river	VIC	A	+	+	+	Gamma	MG764108
Pacific black duck CoV-8907-2016/6/7-LC/VIC	Pacific black duck	7/06/2016	Lake Connewarre	VIC	A	-	-	+	Gamma	MG764109
Pacific black duck CoV-8916-2016/6/7-LC/VIC	Pacific black duck	7/06/2016	Lake Connewarre	VIC	A	-	+	+		
Pacific black duck CoV-9707-2016/12/21-LC/VIC	Pacific black duck	21/12/2016	Lake Connewarre	VIC	A	-	+	+		
Pacific black duck CoV-9709-2016/12/21-LC/VIC	Pacific black duck	21/12/2016	Lake Connewarre	VIC	J	-	+	+		
Pacific black duck CoV-9710-2016/12/21-LC/VIC	Pacific black duck	21/12/2016	Lake Connewarre	VIC	J	+	+	+	Gamma	MG764110
Pacific black duck CoV-9711-2016/12/21-LC/VIC	Pacific black duck	21/12/2016	Lake Connewarre	VIC	J	+	+	+	Gamma	MG764111
Pacific black duck CoV-9712-2016/12/21-LC/VIC	Pacific black duck	21/12/2016	Lake Connewarre	VIC	J	-	+	+		
Pacific black duck CoV-9713-2016/12/21-LC/VIC	Pacific black duck	21/12/2016	Lake Connewarre	VIC	J	+	+	+		
Pacific black duck CoV-9714-2016/12/21-LC/VIC	Pacific black duck	21/12/2016	Lake Connewarre	VIC	A	-	+	+		
Pacific black duck CoV-9715-2016/12/22-LC/VIC	Pacific black duck	22/12/2016	Lake Connewarre	VIC	A	-	+	+		
Pacific black duck CoV-9718-2016/12/22-LC/VIC	Pacific black duck	22/12/2016	Lake Connewarre	VIC	J	-	-	+	Gamma	MG764112
Pacific black duck CoV-9719-2016/12/22-LC/VIC	Pacific black duck	22/12/2016	Lake Connewarre	VIC	A	-	-	+	Gamma	MG764113
Pacific black duck CoV-9720-2016/12/22-LC/VIC	Pacific black duck	22/12/2016	Lake Connewarre	VIC	A	-	+	+		
Pacific black duck CoV-9721-2016/12/22-LC/VIC	Pacific black duck	22/12/2016	Lake Connewarre	VIC	A	-	+	+	Gamma	MG764114
Pacific Black Duck CoV-G0001-2016/12/21-LC/VIC	Pacific Black Duck	21/12/2016	Lake Connewarre	VIC	J	+	+	+	Gamma, Delta	MG764115, MG764116
Pied heron CoV-9518-2016/4/30-HD/NT	Pied heron	30/04/2016	Humpty Doo	NT	A	-	+	+	Delta	MG764117
Pied heron CoV-9521-2016/5/1-HD/NT	Pied heron	1/05/2016	Humpty Doo	NT	A	-	+	-	Delta	MG764118
Pied heron CoV-9522-2016/5/1-HD/NT	Pied heron	1/05/2016	Humpty Doo	NT	J	-	+	-	Delta	MG764119
Pied heron CoV-9523-2016/5/1-HD/NT	Pied heron	1/05/2016	Humpty Doo	NT	J	-	+	+	Delta	MG764120
Pied heron CoV-9524-2016/5/1-HD/NT	Pied heron	1/05/2016	Humpty Doo	NT	J	-	+	+	Delta	MG764121
Radjah shelduck CoV-9515-2016/4/28-HD/NT	Radjah shelduck	28/04/2016	Humpty Doo	NT	A	+	+	+	Gamma	MG764122
Red-necked stint CoV-10034-2017/1/7-YC/VIC	Red-necked stint	7/01/2017	Yallock Creek	VIC	U	-	+	-		
Red-necked stint CoV-10402-2017/1/21-BI/VIC	Red-necked stint	21/01/2017	Barrallier Island	VIC	U	-	+	+	Gamma	MG764123
Red-necked stint CoV-10412-2017/1/21-BI/VIC	Red-necked stint	21/01/2017	Barrallier Island	VIC	U	+	-	-		

Red-necked stint CoV-10415-2017/1/21-BI/VIC	Red-necked stint	21/01/2017	Barrallier Island	VIC	U	+	+	+	Gamma	MG764124
Red-necked stint CoV-10442-2017/1/21-BI/VIC	Red-necked stint	21/01/2017	Barrallier Island	VIC	U	+	-	-		
Red-necked stint CoV-10443-2017/1/21-BI/VIC	Red-necked stint	21/01/2017	Barrallier Island	VIC	U	+	-	-		
Red-necked stint CoV-10452-2017/1/21-BI/VIC	Red-necked stint	21/01/2017	Barrallier Island	VIC	U	+	-	-		
Red-necked stint CoV-10455-2017/1/21-BI/VIC	Red-necked stint	21/01/2017	Barrallier Island	VIC	U	-	-	+		
Red-necked stint CoV-10461-2017/1/21-BI/VIC	Red-necked stint	21/01/2017	Barrallier Island	VIC	U	+	-	-		
Red-necked stint CoV-10467-2017/1/21-BI/VIC	Red-necked stint	21/01/2017	Barrallier Island	VIC	U	-	-	+	Delta	MG764125
Red-necked stint CoV-10479-2017/1/21-BI/VIC	Red-necked stint	21/01/2017	Barrallier Island	VIC	U	-	-	+		
Red-necked stint CoV-10485-2017/1/21-BI/VIC	Red-necked stint	21/01/2017	Barrallier Island	VIC	U	+	-	-		
Red-necked stint CoV-10490-2017/1/21-BI/VIC	Red-necked stint	21/01/2017	Barrallier Island	VIC	U	+	-	-		
Red-necked stint CoV-10496-2017/1/21-BI/VIC	Red-necked stint	21/01/2017	Barrallier Island	VIC	U	+	-	-		
Red-necked stint CoV-10504-2017/1/21-BI/VIC	Red-necked stint	21/01/2017	Barrallier Island	VIC	U	+	-	-		
Red-necked stint CoV-10517-2017/1/21-BI/VIC	Red-necked stint	21/01/2017	Barrallier Island	VIC	U	+	-	-		
Red-necked stint CoV-10519-2017/1/21-BI/VIC	Red-necked stint	21/01/2017	Barrallier Island	VIC	U	+	-	-		
Red-necked stint CoV-10522-2017/1/21-BI/VIC	Red-necked stint	21/01/2017	Barrallier Island	VIC	U	+	-	-		
Red-necked stint CoV-10523-2017/1/21-BI/VIC	Red-necked stint	21/01/2017	Barrallier Island	VIC	U	+	-	-		
Red-necked stint CoV-10524-2017/1/21-BI/VIC	Red-necked stint	21/01/2017	Barrallier Island	VIC	U	+	-	-		
Red-necked stint CoV-10533-2017/1/21-BI/VIC	Red-necked stint	21/01/2017	Barrallier Island	VIC	U	+	-	-		
Red-necked stint CoV-10540-2017/1/21-BI/VIC	Red-necked stint	21/01/2017	Barrallier Island	VIC	U	+	+	+		
Red-necked stint CoV-10554-2017/1/21-BI/VIC	Red-necked stint	21/01/2017	Barrallier Island	VIC	U	+	-	-		
Red-necked stint CoV-10563-2017/1/21-BI/VIC	Red-necked stint	21/01/2017	Barrallier Island	VIC	U	+	-	-		
Red-necked stint CoV-9060-2016/12/30-WS/VIC	Red-necked stint	30/12/2016	Werribee South	VIC	U	-	-	+		
Red-necked stint CoV-9068-2016/12/30-WS/VIC	Red-necked stint	30/12/2016	Werribee South	VIC	U	-	-	+	Gamma	MG764126
Red-necked stint CoV-9074-2016/12/30-WS/VIC	Red-necked stint	30/12/2016	Werribee South	VIC	U	-	+	+		
Red-necked stint CoV-9075-2016/12/30-WS/VIC	Red-necked stint	30/12/2016	Werribee South	VIC	U	+	+	-		
Red-necked stint CoV-9729-2016/12/28-WS/VIC	Red-necked stint	28/12/2016	Werribee South	VIC	U	-	+	-	Gamma	MG764127
Red-necked stint CoV-9736-2016/12/28-WS/VIC	Red-necked stint	28/12/2016	Werribee South	VIC	U	-	-	+		
Red-necked stint CoV-9738-2016/12/28-WS/VIC	Red-necked stint	28/12/2016	Werribee South	VIC	U	-	+	+	Gamma	MG764128
Red-necked stint CoV-9745-2016/12/28-WS/VIC	Red-necked stint	28/12/2016	Werribee South	VIC	U	-	+	+		
Red-necked stint CoV-9752-2016/12/28-WS/VIC	Red-necked stint	28/12/2016	Werribee South	VIC	U	-	+	+	Gamma	MG764129

Red-necked stint CoV-9772-2016/12/28-WS/VIC	Red-necked stint	28/12/2016	Werribee South	VIC	U	-	+	+	Gamma	MG764130
Red-necked stint CoV-9780-2016/12/29-WS/VIC	Red-necked stint	29/12/2016	Werribee South	VIC	U	-	+	-	Gamma	MG764131
Red-necked stint CoV-9786-2016/12/29-WS/VIC	Red-necked stint	29/12/2016	Werribee South	VIC	U	-	+	+	Gamma	MG764132
Red-necked stint CoV-9794-2016/12/29-WS/VIC	Red-necked stint	29/12/2016	Werribee South	VIC	U	-	+	+		
Red-necked stint CoV-9795-2016/12/29-WS/VIC	Red-necked stint	29/12/2016	Werribee South	VIC	U	-	-	+	Delta	MG764133
Red-necked stint CoV-9809-2016/12/29-WS/VIC	Red-necked stint	29/12/2016	Werribee South	VIC	U	-	+	+		
Red-necked stint CoV-9810-2016/12/29-WS/VIC	Red-necked stint	29/12/2016	Werribee South	VIC	U	-	+	+		
Red-necked stint CoV-9811-2016/12/29-WS/VIC	Red-necked stint	29/12/2016	Werribee South	VIC	U	-	+	+		
Red-necked stint CoV-9967-2017/1/7-YC/VIC	Red-necked stint	7/01/2017	Yallock Creek	VIC	U	+	-	-		
Ruddy turnstone CoV-9549-2016/11/18-BB/TAS	Ruddy turnstone	18/11/2016	Burges Bay	TAS	U	+	+	+	Gamma	MG764134
Ruddy turnstone CoV-9550-2016/11/18-BB/TAS	Ruddy turnstone	18/11/2016	Burges Bay	TAS	U	+	+	+	Gamma	MG764135
Ruddy turnstone CoV-9551-2016/11/18-BB/TAS	Ruddy turnstone	18/11/2016	Burges Bay	TAS	U	-	-	+		
Ruddy turnstone CoV-9560-2016/11/18-BB/TAS	Ruddy turnstone	18/11/2016	Burges Bay	TAS	U	-	+	+	Delta	MG764136
Ruddy turnstone CoV-9564-2016/11/18-BB/TAS	Ruddy turnstone	18/11/2016	Burges Bay	TAS	U	+	+	+	Gamma	MG764137
Ruddy turnstone CoV-9566-2016/11/18-BB/TAS	Ruddy turnstone	18/11/2016	Burges Bay	TAS	U	+	+	+	Gamma	MG764138
Ruddy turnstone CoV-9567-2016/11/18-BB/TAS	Ruddy turnstone	18/11/2016	Burges Bay	TAS	U	-	+	+	Delta	MH090081
Ruddy turnstone CoV-9574-2016/11/18-BB/TAS	Ruddy turnstone	18/11/2016	Burges Bay	TAS	U	-	+	-	Delta	MG764139
Ruddy turnstone CoV-9582-2016/11/18-BB/TAS	Ruddy turnstone	18/11/2016	Burges Bay	TAS	U	-	+	+		
Ruddy turnstone CoV-9583-2016/11/18-BB/TAS	Ruddy turnstone	18/11/2016	Burges Bay	TAS	U	-	-	+		
Ruddy turnstone CoV-9586-2016/11/18-BB/TAS	Ruddy turnstone	18/11/2016	Burges Bay	TAS	U	-	-	+		
Ruddy turnstone CoV-9587-2016/11/18-BB/TAS	Ruddy turnstone	18/11/2016	Burges Bay	TAS	U	+	+	+	Gamma	MG764140
Ruddy turnstone CoV-9592-2016/11/18-BB/TAS	Ruddy turnstone	18/11/2016	Burges Bay	TAS	U	-	+	-	Gamma	MG764141
Ruddy turnstone CoV-9596-2016/11/19-CM/TAS	Ruddy turnstone	19/11/2016	Central Manuka	TAS	U	+	+	+	Gamma	MG764142
Ruddy turnstone CoV-9597-2016/11/19-CM/TAS	Ruddy turnstone	19/11/2016	Central Manuka	TAS	U	+	+	+	Gamma	MG764143
Ruddy turnstone CoV-9600-2016/11/19-CM/TAS	Ruddy turnstone	19/11/2016	Central Manuka	TAS	U	+	-	+	Gamma	MG764144
Ruddy turnstone CoV-9602-2016/11/19-CM/TAS	Ruddy turnstone	19/11/2016	Central Manuka	TAS	U	+	+	+	Gamma	MG764145
Ruddy turnstone CoV-9604-2016/11/19-CM/TAS	Ruddy turnstone	19/11/2016	Central Manuka	TAS	U	-	+	+		
Ruddy turnstone CoV-9605-2016/11/19-CM/TAS	Ruddy turnstone	19/11/2016	Central Manuka	TAS	U	-	+	+	Gamma	MH090080
Ruddy turnstone CoV-9610-2016/11/19-CM/TAS	Ruddy turnstone	19/11/2016	Central Manuka	TAS	U	-	+	+	Delta	MG764146
Ruddy turnstone CoV-9612-2016/11/19-CM/TAS	Ruddy turnstone	19/11/2016	Central Manuka	TAS	U	+	+	+	Gamma	MG764147

Ruddy turnstone CoV-9614-2016/11/19-CM/TAS	Ruddy turnstone	19/11/2016	Central Manuka	TAS	U	-	+	Not Tested*		
Ruddy turnstone CoV-9617-2016/11/19-CM/TAS	Ruddy turnstone	19/11/2016	Central Manuka	TAS	U	+	+	+	Gamma	MG764148
Ruddy turnstone CoV-9619-2016/11/19-CM/TAS	Ruddy turnstone	19/11/2016	Central Manuka	TAS	U	-	+	+		
Ruddy turnstone CoV-9623-2016/11/19-CM/TAS	Ruddy turnstone	19/11/2016	Central Manuka	TAS	U	-	+	+	Gamma	MG764149
Ruddy turnstone CoV-9624-2016/11/19-CM/TAS	Ruddy turnstone	19/11/2016	Central Manuka	TAS	U	-	+	+	Gamma	MG764150
Ruddy turnstone CoV-9628-2016/11/22-DW/TAS	Ruddy turnstone	22/11/2016	Dripping Wells	TAS	U	+	+	+	Gamma	MG764151
Ruddy turnstone CoV-9652-2016/11/22-DW/TAS	Ruddy turnstone	22/11/2016	Dripping Wells	TAS	U	+	+	+		
Ruddy turnstone CoV-9653-2016/11/22-DW/TAS	Ruddy turnstone	22/11/2016	Dripping Wells	TAS	U	+	+	+		
Ruddy turnstone CoV-9656-2016/11/27-NV/SA	Ruddy turnstone	27/11/2016	Nene Valley	SA	U	+	+	+		
Ruddy turnstone CoV-9657-2016/11/27-NV/SA	Ruddy turnstone	27/11/2016	Nene Valley	SA	U	+	+	+		
Ruddy turnstone CoV-9662-2016/11/27-NV/SA	Ruddy turnstone	27/11/2016	Nene Valley	SA	U	-	-	+	Gamma	MG764152
Ruddy turnstone CoV-9668-2016/11/27-NV/SA	Ruddy turnstone	27/11/2016	Nene Valley	SA	U	-	-	+	Gamma	MG764153
Ruddy turnstone CoV-9671-2016/11/27-NV/SA	Ruddy turnstone	27/11/2016	Nene Valley	SA	U	+	+	+		
Ruddy turnstone CoV-9682-2016/11/28-BP/SA	Ruddy turnstone	28/11/2016	Boatswain Point	SA	U	+	+	+		
Ruddy turnstone CoV-9684-2016/11/28-BP/SA	Ruddy turnstone	28/11/2016	Boatswain Point	SA	U	+	-	+		
Ruddy turnstone CoV-9685-2016/11/28-BP/SA	Ruddy turnstone	28/11/2016	Boatswain Point	SA	U	+	+	+		
Ruddy turnstone CoV-9686-2016/11/28-BP/SA	Ruddy turnstone	28/11/2016	Boatswain Point	SA	U	-	-	+		
Ruddy turnstone CoV-9687-2016/11/28-BP/SA	Ruddy turnstone	28/11/2016	Boatswain Point	SA	U	+	+	+		
Ruddy turnstone CoV-9690-2016/11/28-BP/SA	Ruddy turnstone	28/11/2016	Boatswain Point	SA	U	+	+	+		
Ruddy turnstone CoV-9691-2016/11/28-BP/SA	Ruddy turnstone	28/11/2016	Boatswain Point	SA	U	+	+	+		
Ruddy turnstone CoV-9692-2016/11/28-BP/SA	Ruddy turnstone	28/11/2016	Boatswain Point	SA	U	+	+	+		
Ruddy turnstone CoV-9693-2016/11/28-BP/SA	Ruddy turnstone	28/11/2016	Boatswain Point	SA	U	-	+	+		
Ruddy turnstone CoV-9694-2016/11/28-BP/SA	Ruddy turnstone	28/11/2016	Boatswain Point	SA	U	+	+	+	Gamma	MG764154
Ruddy turnstone CoV-9696-2016/11/28-BP/SA	Ruddy turnstone	28/11/2016	Boatswain Point	SA	U	+	+	+	Gamma	MG764155
Ruddy turnstone CoV-9698-2016/11/28-BP/SA	Ruddy turnstone	28/11/2016	Boatswain Point	SA	U	-	+	+	Delta	MG764156
Ruddy turnstone CoV-9699-2016/11/28-BP/SA	Ruddy turnstone	28/11/2016	Boatswain Point	SA	U	+	+	+		
Wood duck CoV-10220-2016/6/13-CR/VIC	Wood duck	13/06/2016	Carlisle river	VIC	A	+	-	-		

*Due to insufficient RNA for assay

Supplementary Figure S1. *Maximum likelihood phylogenetic tree of the 277 bp fragment of the polymerase gene of gammacoronaviruses from wild birds including the sequences obtained in this study. Bootstrap confidence of each branch was calculated from 1000 replicates. The region where each sequence was obtained is indicated with colour. Australia (red), Madagascar (teal), China, Hong Kong and Korea (dark blue), Bering Strait region (green), United States of America (light blue) and Sweden (pink). The sampling location and state of each Australian sample is identified with a two letter code and the state: New South Wales (NSW): MM-Moulamein; Northern Territory (NT): HD-Humpty Doo; South Australia (SA): BP-Boatswain Point, NV-Nene Valley; Tasmania (TAS): BB-Borges Bay, King Island, CM-Central Manuka, King Island, DW-Dripping Wells, King Island, Tasmania; Victoria (VIC): BI-Barrallier Island, CR-Carlisle River, LC-Lake Connewarre, PV-Paynesville, WS-Werribee South.*

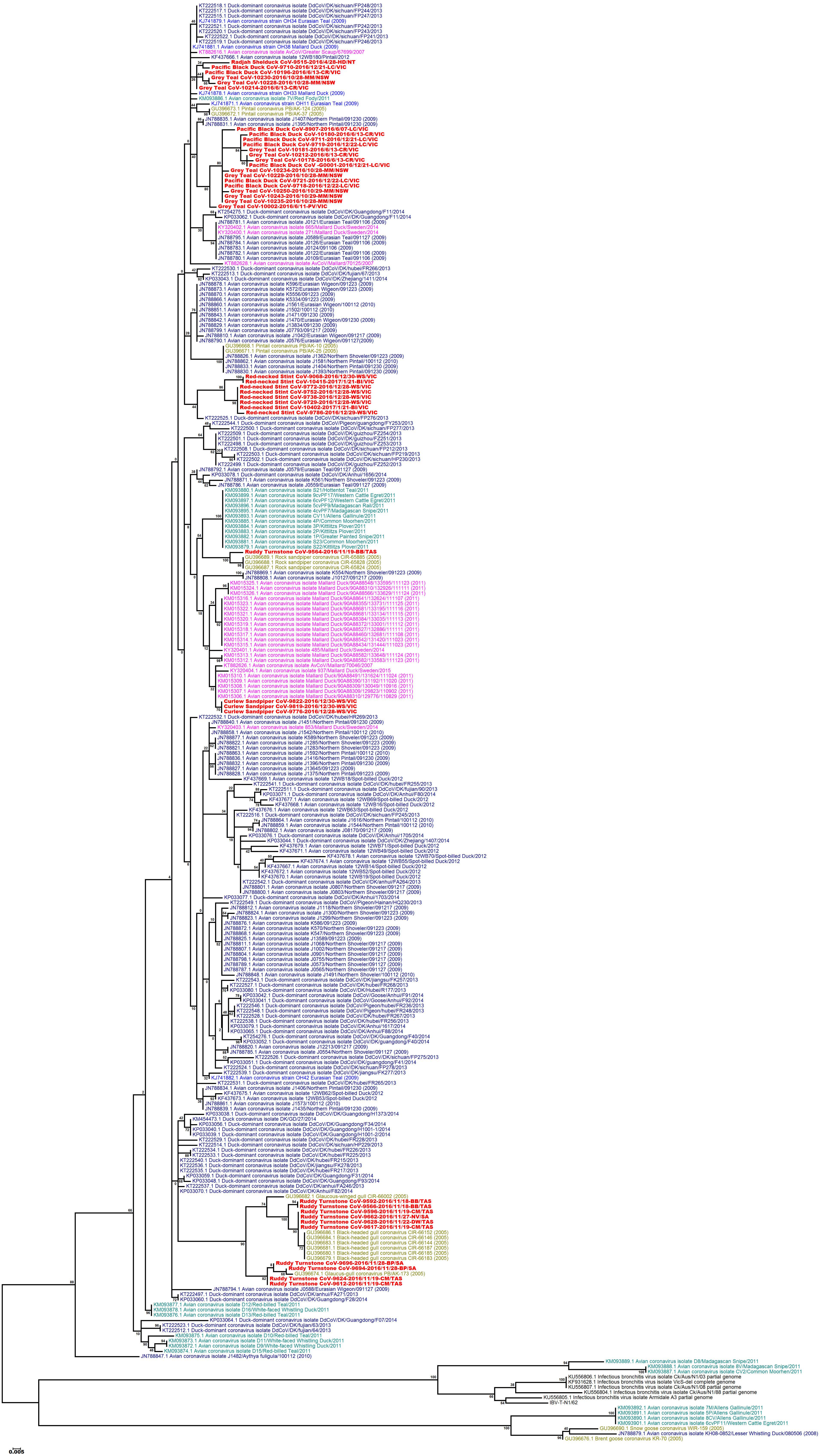

KT222518.1 Duck-dominant coronavirus isolate DdCoV/DK/sichuan/FP248/2013
KT222517.1 Duck-dominant coronavirus isolate DdCoV/DK/sichuan/FP244/2013
KT222515.1 Duck-dominant coronavirus isolate DdCoV/DK/sichuan/FP247/2013
KJ741879.1 Avian coronavirus strain OH34 Eurasian Teal (2009)
KT222521.1 Duck-dominant coronavirus isolate DdCoV/DK/sichuan/FP242/2013
KT222520.1 Duck-dominant coronavirus isolate DdCoV/DK/sichuan/FP243/2013
KT222522.1 Duck-dominant coronavirus isolate DdCoV/DK/sichuan/FP241/2013
KT222519.1 Duck-dominant coronavirus isolate DdCoV/DK/sichuan/FP246/2013
KJ741881.1 Avian coronavirus strain OH38 Mallard Duck (2009)
KT882616.1 Avian coronavirus isolate AvCoV/Greater Scaup/67699/2007
KF437666.1 Avian coronavirus isolate 12WB180/Pintail/2012
Radjah Shelduck CoV-9515-2016/4/28-HD/NT
Pacific Black Duck CoV-9710-2016/12/21-LC/VIC
Pacific Black Duck CoV-10196-2016/6/13-CR/VIC
Grey Teal CoV-10230-2016/10/28-MM/NSW
Grey Teal CoV-10228-2016/10/28-MM/NSW
Grey Teal CoV-10214-2016/6/13-CR/VIC
KJ741878.1 Avian coronavirus strain OH33 Mallard Duck (2009)
KM093886.1 Avian coronavirus isolate 7V/Red Fody/2011
KJ741871.1 Avian coronavirus strain OH11 Eurasian Teal (2009)
GU396673.1 Pintail coronavirus PB/AK-124 (2005)
GU396672.1 Pintail coronavirus PB/AK-37 (2005)
JN788835.1 Avian coronavirus isolate J1407/Northern Pintail/091230 (2009)
JN788831.1 Avian coronavirus isolate J1395/Northern Pintail/091230 (2009)
Pacific Black Duck CoV-8907-2016/6/07-LC/VIC
Pacific Black Duck CoV-10180-2016/6/13-CR/VIC
Pacific Black Duck CoV-9711-2016/12/21-LC/VIC
Pacific Black Duck CoV-9719-2016/12/22-LC/VIC
Grey Teal CoV-10181-2016/6/13-CR/VIC
Grey Teal CoV-10212-2016/6/13-CR/VIC
Grey Teal CoV-10178-2016/6/13-CR/VIC
Pacific Black Duck CoV -G0001-2016/12/21-LC/VIC
Grey Teal CoV-10234-2016/10/28-MM/NSW
Grey Teal CoV-10229-2016/10/28-MM/NSW
Pacific Black Duck CoV-9721-2016/12/22-LC/VIC
Pacific Black Duck CoV-9718-2016/12/22-LC/VIC
Grey Teal CoV-10250-2016/10/29-MM/NSW
Grey Teal CoV-10243-2016/10/29-MM/NSW
Grey Teal CoV-10235-2016/10/28-MM/NSW
Grey Teal CoV-10002-2016/6/11-PV/VIC
KT254275.1 Duck-dominant coronavirus isolate DdCoV/DK/Guangdong/F11/2014
KP033062.1 Duck-dominant coronavirus isolate DdCoV/DK/Guangdong/F11/2014
JN788781.1 Avian coronavirus isolate J0121/Eurasian Teal/091106 (2009)
KY320402.1 Avian coronavirus isolate 665/Mallard Duck/Sweden/2014
KY320400.1 Avian coronavirus isolate 271/Mallard Duck/Sweden/2014
JN788795.1 Avian coronavirus isolate J0589/Eurasian Teal/091127 (2009)
JN788784.1 Avian coronavirus isolate J0126/Eurasian Teal/091106 (2009)
JN788783.1 Avian coronavirus isolate J0124/091106 (2009)
JN788782.1 Avian coronavirus isolate J0122/Eurasian Teal/091106 (2009)
JN788780.1 Avian coronavirus isolate J0109/Eurasian Teal/091106 (2009)
KT882628.1 Avian coronavirus isolate AvCoV/Mallard/70125/2007
KT222530.1 Duck-dominant coronavirus isolate DdCoV/DK/hubei/FR266/2013
KT222513.1 Duck-dominant coronavirus isolate DdCoV/DK/fujian/67/2013
KP033043.1 Duck-dominant coronavirus isolate DdCoV/DK/Zhejiang/1411/2014
JN788878.1 Avian coronavirus isolate K596/Eurasian Wigeon/091223 (2009)
JN788873.1 Avian coronavirus isolate K572/Eurasian Wigeon/091223 (2009)
JN788870.1 Avian coronavirus isolate K5556/091223 (2009)
JN788866.1 Avian coronavirus isolate K5334/091223 (2009)
JN788860.1 Avian coronavirus isolate J1561/Eurasian Wigeon/100112 (2010)
JN788851.1 Avian coronavirus isolate J1502/100112 (2010)
JN788843.1 Avian coronavirus isolate J1471/091230 (2009)
JN788842.1 Avian coronavirus isolate J1470/Eurasian Wigeon/091230 (2009)
JN788829.1 Avian coronavirus isolate J13834/091230 (2009)
JN788799.1 Avian coronavirus isolate J07793/091217 (2009)
JN788810.1 Avian coronavirus isolate J1042/Eurasian Wigeon/091217 (2009)
JN788790.1 Avian coronavirus isolate J0576/Eurasian Wigeon/091127(2009)
GU396668.1 Pintail coronavirus PB/AK-10 (2005)
GU396671.1 Pintail coronavirus PB/AK-25 (2005)
JN788826.1 Avian coronavirus isolate J1362/Northern Shoveler/091223 (2009)
JN788862.1 Avian coronavirus isolate J1581/Northern Pintail/100112 (2010)
JN788833.1 Avian coronavirus isolate J1404/Northern Pintail/091230 (2009)
JN788830.1 Avian coronavirus isolate J1393/Northern Pintail/091230 (2009)
Red-necked Stint CoV-9068-2016/12/30-WS/VIC
Red-necked Stint CoV-10415-2017/1/21-BI/VIC
Red-necked Stint CoV-9772-2016/12/28-WS/VIC
Red-necked Stint CoV-9752-2016/12/28-WS/VIC
Red-necked Stint CoV-9738-2016/12/28-WS/VIC
Red-necked Stint CoV-9729-2016/12/28-WS/VIC
Red-necked Stint CoV-10402-2017/1/21-BI/VIC
Red-necked Stint CoV-9786-2016/12/29-WS/VIC
KT222525.1 Duck-dominant coronavirus isolate DdCoV/DK/sichuan/FP276/2013
KT222544.1 Duck-dominant coronavirus isolate DdCoV/Pigeon/guangdong/FY253/2013
KT222500.1 Duck-dominant coronavirus isolate DdCoV/DK/sichuan/FP277/2013
KT222509.1 Duck-dominant coronavirus isolate DdCoV/DK/guizhou/FZ254/2013
KT222501.1 Duck-dominant coronavirus isolate DdCoV/DK/guizhou/FZ251/2013
KT222498.1 Duck-dominant coronavirus isolate DdCoV/DK/guizhou/FZ253/2013
KT222508.1 Duck-dominant coronavirus isolate DdCoV/DK/sichuan/FP212/2013
KT222503.1 Duck-dominant coronavirus isolate DdCoV/DK/sichuan/FP219/2013
KT222502.1 Duck-dominant coronavirus isolate DdCoV/DK/sichuan/HP230/2013
KT222499.1 Duck-dominant coronavirus isolate DdCoV/DK/guizhou/FZ252/2013
JN788792.1 Avian coronavirus isolate J0579/Eurasian Teal/091127 (2009)
KP033078.1 Duck-dominant coronavirus isolate DdCoV/DK/Anhui/1656/2014
JN788871.1 Avian coronavirus isolate K561/Northern Shoveler/091223 (2009)
JN788786.1 Avian coronavirus isolate J0559/Eurasian Teal/091127 (2009)
KM093880.1 Avian coronavirus isolate S21/Hottentot Teal/2011
KM093899.1 Avian coronavirus isolate 9cvPF17/Western Cattle Egret/2011
KM093897.1 Avian coronavirus isolate 6cvPF12/Western Cattle Egret/2011
KM093896.1 Avian coronavirus isolate 5cvPF9/Madagascan Rail/2011
KM093895.1 Avian coronavirus isolate 4cvPF7/Madagascan Snipe/2011
KM093893.1 Avian coronavirus isolate CV11/Allens Gallinule/2011
KM093885.1 Avian coronavirus isolate 4P/Common Moorhen/2011
KM093884.1 Avian coronavirus isolate 3P/Kittlitzs Plover/2011
KM093883.1 Avian coronavirus isolate 2P/Kittlitzs Plover/2011
KM093882.1 Avian coronavirus isolate 1P/Greater Painted Snipe/2011
KM093881.1 Avian coronavirus isolate S23/Common Moorhen/2011
KM093879.1 Avian coronavirus isolate S22/Kittlitzs Plover/2011
Ruddy Turnstone CoV-9564-2016/11/19-BB/TAS
GU396689.1 Rock sandpiper coronavirus CIR-65885 (2005)
GU396688.1 Rock sandpiper coronavirus CIR-65828 (2005)
GU396687.1 Rock sandpiper coronavirus CIR-65824 (2005)
JN788869.1 Avian coronavirus isolate K554/Northern Shoveler/091223 (2009)
JN788808.1 Avian coronavirus isolate J10127/091217 (2009)
KM015325.1 Avian coronavirus isolate Mallard Duck/90A88548/133595/111123 (2011)
KM015324.1 Avian coronavirus isolate Mallard Duck/90A88310/132926/111111 (2011)
KM015326.1 Avian coronavirus isolate Mallard Duck/90A88566/133629/111124 (2011)
KM015316.1 Avian coronavirus isolate Mallard Duck/90A88641/132624/111107 (2011)
KM015323.1 Avian coronavirus isolate Mallard Duck/90A88355/133731/111125 (2011)
KM015322.1 Avian coronavirus isolate Mallard Duck/90A88681/133195/111116 (2011)
KM015321.1 Avian coronavirus isolate Mallard Duck/90A88681/133134/111115 (2011)
KM015320.1 Avian coronavirus isolate Mallard Duck/90A88384/133035/111113 (2011)
KM015319.1 Avian coronavirus isolate Mallard Duck/90A88372/133001/111112 (2011)
KM015318.1 Avian coronavirus isolate Mallard Duck/90A88527/132886/111111 (2011)
KM015317.1 Avian coronavirus isolate Mallard Duck/90A88460/132681/111108 (2011)
KM015314.1 Avian coronavirus isolate Mallard Duck/90A88542/131420/111023 (2011)
KM015315.1 Avian coronavirus isolate Mallard Duck/90A88434/131444/111023 (2011)
KY320401.1 Avian coronavirus isolate 485/Mallard Duck/Sweden/2014
KM015313.1 Avian coronavirus isolate Mallard Duck/90A88582/133648/111124 (2011)
KM015312.1 Avian coronavirus isolate Mallard Duck/90A88582/133583/111123 (2011)
KT882626.1 Avian coronavirus isolate AvCoV/Mallard/70046/2007
KY320404.1 Avian coronavirus isolate 937/Mallard Duck/Sweden/2015
KM015310.1 Avian coronavirus isolate Mallard Duck/90A88491/131624/111024 (2011)
KM015309.1 Avian coronavirus isolate Mallard Duck/90A88390/131192/111020 (2011)
KM015308.1 Avian coronavirus isolate Mallard Duck/90A88309/130049/110916 (2011)
KM015307.1 Avian coronavirus isolate Mallard Duck/90A88310/129823/110902 (2011)
KM015306.1 Avian coronavirus isolate Mallard Duck/90A88310/129776/110829 (2011)
Curlew Sandpiper CoV-9822-2016/12/30-WS/VIC
Curlew Sandpiper CoV-9819-2016/12/30-WS/VIC
Curlew Sandpiper CoV-9776-2016/12/28-WS/VIC
KT222532.1 Duck-dominant coronavirus isolate DdCoV/DK/hubei/HR269/2013
JN788840.1 Avian coronavirus isolate J1451/Northern Pintail/091230 (2009)
KY320403.1 Avian coronavirus isolate 853/Mallard Duck/Sweden/2014
JN788858.1 Avian coronavirus isolate J1542/Northern Pintail/100112 (2010)
JN788877.1 Avian coronavirus isolate K589/Northern Shoveler/091223 (2009)
JN788822.1 Avian coronavirus isolate J1285/Northern Shoveler/091223 (2009)
JN788821.1 Avian coronavirus isolate J1283/Northern Shoveler/091223 (2009)
JN788863.1 Avian coronavirus isolate J1592/Northern Pintail/100112 (2010)
JN788836.1 Avian coronavirus isolate J1416/Northern Pintail/091230 (2009)
JN788832.1 Avian coronavirus isolate J1396/Northern Pintail/091230 (2009)
JN788827.1 Avian coronavirus isolate J13645/091223 (2009)
JN788828.1 Avian coronavirus isolate J1375/Northern Pintail/091223 (2009)
KF437669.1 Avian coronavirus isolate 12WB18/Spot-billed Duck/2012
KT222541.1 Duck-dominant coronavirus isolate DdCoV/DK/hubei/FR255/2013
KT222511.1 Duck-dominant coronavirus isolate DdCoV/DK/fujian/90/2013
KP033071.1 Duck-dominant coronavirus isolate DdCoV/DK/Anhui/F80/2014
KF437677.1 Avian coronavirus isolate 12WB69/Spot-billed Duck/2012
KF437668.1 Avian coronavirus isolate 12WB16/Spot-billed Duck/2012
KF437676.1 Avian coronavirus isolate 12WB63/Spot-billed Duck/2012
KT222516.1 Duck-dominant coronavirus isolate DdCoV/DK/sichuan/FP245/2013
JN788864.1 Avian coronavirus isolate J1616/Northern Pintail/100112 (2010)
JN788859.1 Avian coronavirus isolate J1544/Northern Pintail/100112 (2010)
JN788802.1 Avian coronavirus isolate J08170/091217 (2009)
KP033076.1 Duck-dominant coronavirus isolate DdCoV/DK/Anhui/1705/2014
KP033044.1 Duck-dominant coronavirus isolate DdCoV/DK/Zhejiang/1407/2014
KF437679.1 Avian coronavirus isolate 12WB71/Spot-billed Duck/2012
KF437671.1 Avian coronavirus isolate 12WB49/Spot-billed Duck/2012
KF437678.1 Avian coronavirus isolate 12WB70/Spot-billed Duck/2012
KF437674.1 Avian coronavirus isolate 12WB55/Spot-billed Duck/2012
KF437667.1 Avian coronavirus isolate 12WB14/Spot-billed Duck/2012
KF437672.1 Avian coronavirus isolate 12WB52/Spot-billed Duck/2012
KF437670.1 Avian coronavirus isolate 12WB19/Spot-billed Duck/2012
KT222542.1 Duck-dominant coronavirus isolate DdCoV/DK/anhui/FA264/2013
JN788801.1 Avian coronavirus isolate J0807/Northern Shoveler/091217 (2009)
JN788800.1 Avian coronavirus isolate J0803/Northern Shoveler/091217 (2009)
KP033077.1 Duck-dominant coronavirus isolate DdCoV/DK/Anhui/1703/2014
KT222549.1 Duck-dominant coronavirus isolate DdCoV/Pigeon/Hainan/HQ230/2013
JN788812.1 Avian coronavirus isolate J1118/Northern Shoveler/091217 (2009)
JN788824.1 Avian coronavirus isolate J1300/Northern Shoveler/091223 (2009)
JN788823.1 Avian coronavirus isolate J1299/Northern Shoveler/091223 (2009)
JN788876.1 Avian coronavirus isolate K586/091223 (2009)
JN788872.1 Avian coronavirus isolate K570/Northern Shoveler/091223 (2009)
JN788868.1 Avian coronavirus isolate K547/Northern Shoveler/091223 (2009)
JN788825.1 Avian coronavirus isolate J13589/091223 (2009)
JN788811.1 Avian coronavirus isolate J1068/Northern Shoveler/091217 (2009)
JN788807.1 Avian coronavirus isolate J1002/Northern Shoveler/091217 (2009)
JN788804.1 Avian coronavirus isolate J0901/Northern Shoveler/091217 (2009)
JN788798.1 Avian coronavirus isolate J0755/Northern Shoveler/091217 (2009)
JN788789.1 Avian coronavirus isolate J0573/Northern Shoveler/091127 (2009)
JN788787.1 Avian coronavirus isolate J0565/Northern Shoveler/091127 (2009)
JN788848.1 Avian coronavirus isolate J1491/Northern Shoveler/100112 (2010)
KT222543.1 Duck-dominant coronavirus isolate DdCoV/DK/jiangsu/FK257/2013
KT222527.1 Duck-dominant coronavirus isolate DdCoV/DK/hubei/FR268/2013
KP033080.1 Duck-dominant coronavirus isolate DdCoV/DK/Hubei/R177/2013
KP033042.1 Duck-dominant coronavirus isolate DdCoV/Goose/Anhui/F91/2014
KP033041.1 Duck-dominant coronavirus isolate DdCoV/Goose/Anhui/F92/2014
KT222546.1 Duck-dominant coronavirus isolate DdCoV/Pigeon/hubei/FR236/2013
KT222548.1 Duck-dominant coronavirus isolate DdCoV/Pigeon/hubei/FR248/2013
KT222528.1 Duck-dominant coronavirus isolate DdCoV/DK/hubei/FR267/2013
KT222538.1 Duck-dominant coronavirus isolate DdCoV/DK/hubei/FR256/2013
KP033079.1 Duck-dominant coronavirus isolate DdCoV/DK/Anhui/1617/2014
KP033065.1 Duck-dominant coronavirus isolate DdCoV/DK/Anhui/F88/2014
KT254276.1 Duck-dominant coronavirus isolate DdCoV/DK/Guangdong/F40/2014
KP033052.1 Duck-dominant coronavirus isolate DdCoV/DK/guangdong/F40/2014
JN788820.1 Avian coronavirus isolate J12213/091217 (2009)
JN788785.1 Avian coronavirus isolate J0554/Northern Shoveler/091127 (2009)
KT222526.1 Duck-dominant coronavirus isolate DdCoV/DK/sichuan/FP275/2013
KP033051.1 Duck-dominant coronavirus isolate DdCoV/DK/guangdong/F41/2014
KT222524.1 Duck-dominant coronavirus isolate DdCoV/DK/sichuan/FP278/2013
KT222539.1 Duck-dominant coronavirus isolate DdCoV/DK/jiangsu/FK277/2013
KJ741882.1 Avian coronavirus strain OH42 Eurasian Teal (2009)
KT222531.1 Duck-dominant coronavirus isolate DdCoV/DK/hubei/FR265/2013
JN788834.1 Avian coronavirus isolate J1406/Northern Pintail/091230 (2009)
KF437675.1 Avian coronavirus isolate 12WB62/Spot-billed Duck/2012
KF437673.1 Avian coronavirus isolate 12WB53/Spot-billed Duck/2012
JN788861.1 Avian coronavirus isolate J1573/100112 (2010)
JN788839.1 Avian coronavirus isolate J1435/Northern Pintail/091230 (2009)
KP033038.1 Duck-dominant coronavirus isolate DdCoV/DK/Guangdong/H1373/2014
KM454473.1 Duck coronavirus isolate DK/GD/27/2014
KP033056.1 Duck-dominant coronavirus isolate DdCoV/DK/Guangdong/F34/2014
KP033040.1 Duck-dominant coronavirus isolate DdCoV/DK/Guangdong/H1001-1/2014
KP033039.1 Duck-dominant coronavirus isolate DdCoV/DK/Guangdong/H1001-2/2014
KT222529.1 Duck-dominant coronavirus isolate DdCoV/DK/hubei/FR228/2013
KT222514.1 Duck-dominant coronavirus isolate DdCoV/DK/sichuan/HP229/2013
KT222534.1 Duck-dominant coronavirus isolate DdCoV/DK/hubei/FR226/2013
KT222533.1 Duck-dominant coronavirus isolate DdCoV/DK/hubei/FR225/2013
KT222540.1 Duck-dominant coronavirus isolate DdCoV/DK/hubei/FR215/2013
KT222536.1 Duck-dominant coronavirus isolate DdCoV/DK/jiangsu/FK278/2013
KT222535.1 Duck-dominant coronavirus isolate DdCoV/DK/hubei/FR217/2013
KP033059.1 Duck-dominant coronavirus isolate DdCoV/DK/Guangdong/F31/2014
KP033048.1 Duck-dominant coronavirus isolate DdCoV/DK/Guangdong/F93/2014
KT222537.1 Duck-dominant coronavirus isolate DdCoV/DK/anhui/FA246/2013
KP033070.1 Duck-dominant coronavirus isolate DdCoV/DK/Anhui/F82/2014
GU396682.1 Glaucous-winged gull CIR-66002 (2005)
Ruddy Turnstone CoV-9592-2016/11/18-BB/TAS
Ruddy Turnstone CoV-9566-2016/11/18-BB/TAS
Ruddy Turnstone CoV-9596-2016/11/19-CM/TAS
Ruddy Turnstone CoV-9662-2016/11/27-NV/SA
Ruddy Turnstone CoV-9628-2016/11/22-DW/TAS
Ruddy Turnstone CoV-9617-2016/11/19-CM/TAS
GU396686.1 Black-headed gull coronavirus CIR-66152 (2005)
GU396684.1 Black-headed gull coronavirus CIR-66146 (2005)
GU396683.1 Black-headed gull coronavirus CIR-66144 (2005)
GU396681.1 Black-headed gull coronavirus CIR-66187 (2005)
GU396680.1 Black-headed gull coronavirus CIR-66185 (2005)
GU396679.1 Black-headed gull coronavirus CIR-66183 (2005)
Ruddy Turnstone CoV-9696-2016/11/28-BP/SA
Ruddy Turnstone CoV-9694-2016/11/28-BP/SA
GU396674.1 Glaucous-gull coronavirus PB/AK-173 (2005)
Ruddy Turnstone CoV-9624-2016/11/19-CM/TAS
Ruddy Turnstone CoV-9612-2016/11/19-CM/TAS
JN788794.1 Avian coronavirus isolate J0588/Eurasian Wigeon/091127 (2009)
KT222497.1 Duck-dominant coronavirus isolate DdCoV/DK/anhui/FA271/2013
KP033060.1 Duck-dominant coronavirus isolate DdCoV/DK/Guangdong/F28/2014
KM093877.1 Avian coronavirus isolate D12/Red-billed Teal/2011
KM093878.1 Avian coronavirus isolate D16/White-faced Whistling Duck/2011
KM093876.1 Avian coronavirus isolate D13/Red-billed Teal/2011
KP033064.1 Duck-dominant coronavirus isolate DdCoV/DK/Guangdong/F07/2014
KT222523.1 Duck-dominant coronavirus isolate DdCoV/DK/fujian/63/2013
KT222512.1 Duck-dominant coronavirus isolate DdCoV/DK/fujian/64/2013
KM093875.1 Avian coronavirus isolate D10/Red-billed Teal/2011
KM093873.1 Avian coronavirus isolate D11/White-faced Whistling Duck/2011
KM093872.1 Avian coronavirus isolate D9/White-faced Whistling Duck/2011
KM093874.1 Avian coronavirus isolate D15/Red-billed Teal/2011
JN788847.1 Avian coronavirus isolate J1482/Aythya fuligula/100112 (2010)
KM093889.1 Avian coronavirus isolate D8/Madagascan Snipe/2011
KM093888.1 Avian coronavirus isolate 8V/Madagascan Snipe/2011
KM093887.1 Avian coronavirus isolate CV2/Common Moorhen/2011
KU556806.1 Infectious bronchitis virus isolate Ck/Aus/N1/03 partial genome
KF931628.1 Infectious bronchitis virus isolate VicS-del complete genome
KU556807.1 Infectious bronchitis virus isolate Ck/Aus/N1/08 partial genome
KU556804.1 Infectious bronchitis virus isolate Ck/Aus/N1/88 partial genome
KU556805.1 Infectious bronchitis virus isolate Armidale A3 partial genome
IBV-T-N1/62
KM093892.1 Avian coronavirus isolate 7M/Allens Gallinule/2011
KM093891.1 Avian coronavirus isolate 5P/Allens Gallinule/2011
KM093890.1 Avian coronavirus isolate 8CV/Allens Gallinule/2011
KM093901.1 Avian coronavirus isolate 6cvPF11/Western Cattle Egret/2011
GU396690.1 Snow goose coronavirus WIR-159 (2005)
JN788879.1 Avian coronavirus isolate KH08-0852/Lesser Whistling Duck/080506 (2008)
GU396676.1 Brent goose coronavirus KR-70 (2005)
0.005

Supplementary Figure S2. *Maximum likelihood phylogenetic tree of the 92 amino acid sequence of the gammacoronaviruses from wild birds. Bootstrap confidence of each branch was calculated from 500 replicates. The region where each sequence was obtained is indicated with colour. Australia (red), Madagascar (teal), China, Hong Kong and Korea (dark blue), Bering Strait region (green), United States of America (light blue) and Sweden (pink). The sampling location and state of each Australian sample is identified with a two letter code and the state: New South Wales (NSW): MM-Moulamein; Northern Territory (NT): HD-Humpty Doo; South Australia (SA): BP-Boatswain Point, NV-Nene Valley; Tasmania (TAS): BB-Borges Bay, King Island, CM-Central Manuka, King Island, DW-Dripping Wells, King Island, Tasmania; Victoria (VIC): BI-Barrallier Island, CR-Carlisle River, LC-Lake Connewarre, PV-Paynesville, WS-Werribee South.*

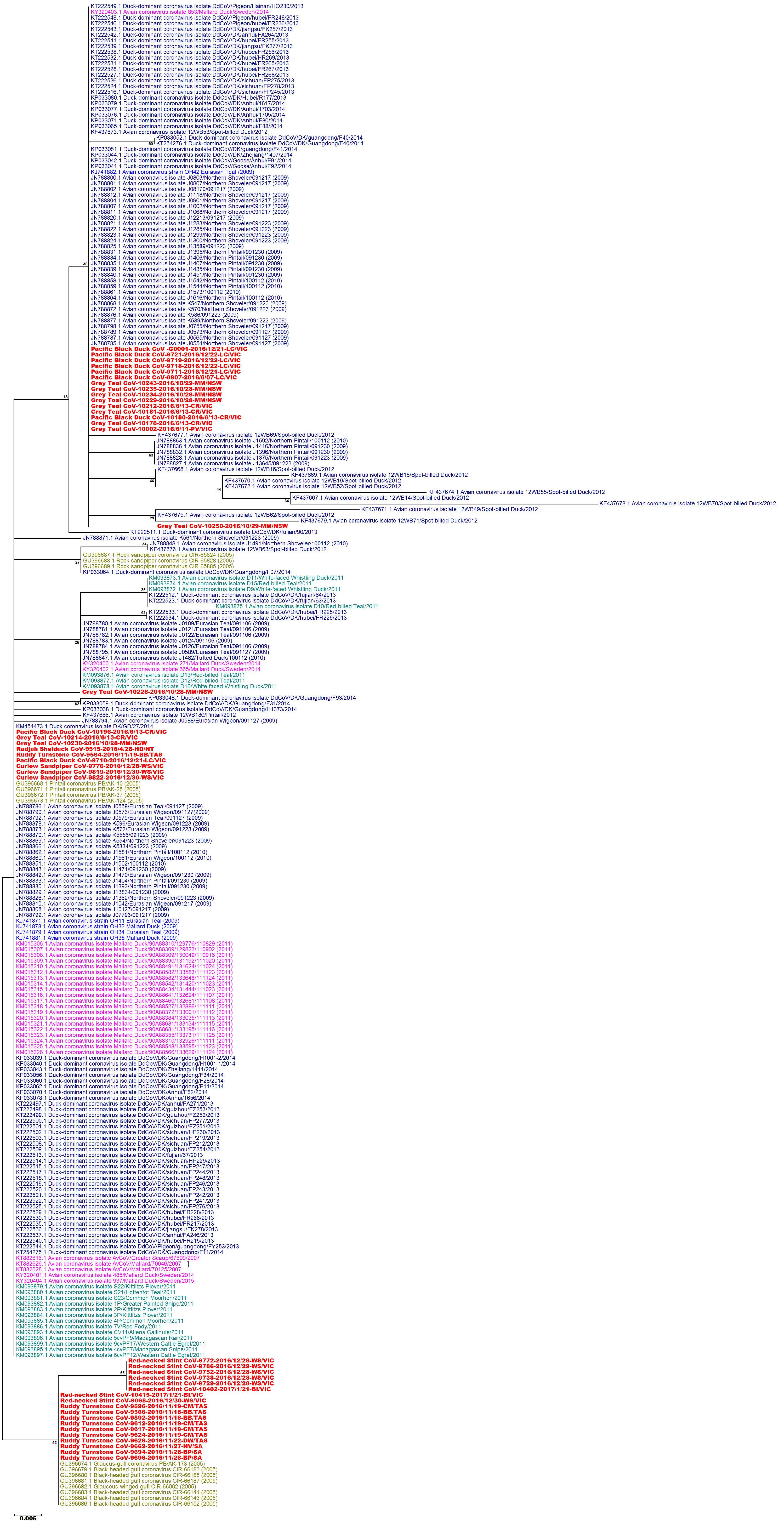

KT222549.1 Duck-dominant coronavirus isolate DdCoV/Pigeon/Hainan/HQ230/2013
KY320403.1 Avian coronavirus isolate 853/Mallard Duck/Sweden/2014
KT222548.1 Duck-dominant coronavirus isolate DdCoV/Pigeon/hubei/FR248/2013
KT222546.1 Duck-dominant coronavirus isolate DdCoV/Pigeon/hubei/FR236/2013
KT222543.1 Duck-dominant coronavirus isolate DdCoV/DK/jiangsu/FK257/2013
KT222542.1 Duck-dominant coronavirus isolate DdCoV/DK/anhui/FA264/2013
KT222541.1 Duck-dominant coronavirus isolate DdCoV/DK/hubei/FR255/2013
KT222539.1 Duck-dominant coronavirus isolate DdCoV/DK/jiangsu/FK277/2013
KT222538.1 Duck-dominant coronavirus isolate DdCoV/DK/hubei/FR256/2013
KT222532.1 Duck-dominant coronavirus isolate DdCoV/DK/hubei/HR269/2013
KT222531.1 Duck-dominant coronavirus isolate DdCoV/DK/hubei/FR265/2013
KT222528.1 Duck-dominant coronavirus isolate DdCoV/DK/hubei/FR267/2013
KT222527.1 Duck-dominant coronavirus isolate DdCoV/DK/hubei/FR268/2013
KT222526.1 Duck-dominant coronavirus isolate DdCoV/DK/sichuan/FP275/2013
KT222524.1 Duck-dominant coronavirus isolate DdCoV/DK/sichuan/FP278/2013
KT222516.1 Duck-dominant coronavirus isolate DdCoV/DK/sichuan/FP245/2013
KP033080.1 Duck-dominant coronavirus isolate DdCoV/DK/Hubei/R177/2013
KP033079.1 Duck-dominant coronavirus isolate DdCoV/DK/Anhui/1617/2014
KP033077.1 Duck-dominant coronavirus isolate DdCoV/DK/Anhui/1703/2014
KP033076.1 Duck-dominant coronavirus isolate DdCoV/DK/Anhui/1705/2014
KP033071.1 Duck-dominant coronavirus isolate DdCoV/DK/Anhui/F80/2014
KP033065.1 Duck-dominant coronavirus isolate DdCoV/DK/Anhui/F88/2014
KF437673.1 Avian coronavirus isolate 12WB53/Spot-billed Duck/2012
KP033052.1 Duck-dominant coronavirus isolate DdCoV/DK/guangdong/F40/2014
60
KT254276.1 Duck-dominant coronavirus isolate DdCoV/DK/Guangdong/F40/2014
KP033051.1 Duck-dominant coronavirus isolate DdCoV/DK/guangdong/F41/2014
KP033044.1 Duck-dominant coronavirus isolate DdCoV/DK/Zhejiang/1407/2014
KP033042.1 Duck-dominant coronavirus isolate DdCoV/Goose/Anhui/F91/2014
KP033041.1 Duck-dominant coronavirus isolate DdCoV/Goose/Anhui/F92/2014
KJ741882.1 Avian coronavirus strain OH42 Eurasian Teal (2009)
JN788800.1 Avian coronavirus isolate J0803/Northern Shoveler/091217 (2009)
JN788801.1 Avian coronavirus isolate J0807/Northern Shoveler/091217 (2009)
JN788802.1 Avian coronavirus isolate J08170/091217 (2009)
JN788812.1 Avian coronavirus isolate J1118/Northern Shoveler/091217 (2009)
JN788804.1 Avian coronavirus isolate J0901/Northern Shoveler/091217 (2009)
JN788807.1 Avian coronavirus isolate J1002/Northern Shoveler/091217 (2009)
JN788811.1 Avian coronavirus isolate J1068/Northern Shoveler/091217 (2009)
JN788820.1 Avian coronavirus isolate J12213/091217 (2009)
JN788821.1 Avian coronavirus isolate J1283/Northern Shoveler/091223 (2009)
JN788822.1 Avian coronavirus isolate J1285/Northern Shoveler/091223 (2009)
JN788823.1 Avian coronavirus isolate J1299/Northern Shoveler/091223 (2009)
JN788824.1 Avian coronavirus isolate J1300/Northern Shoveler/091223 (2009)
JN788825.1 Avian coronavirus isolate J13589/091223 (2009)
30
JN788831.1 Avian coronavirus isolate J1395/Northern Pintail/091230 (2009)
JN788834.1 Avian coronavirus isolate J1406/Northern Pintail/091230 (2009)
JN788835.1 Avian coronavirus isolate J1407/Northern Pintail/091230 (2009)
JN788839.1 Avian coronavirus isolate J1435/Northern Pintail/091230 (2009)
JN788840.1 Avian coronavirus isolate J1451/Northern Pintail/091230 (2009)
JN788858.1 Avian coronavirus isolate J1542/Northern Pintail/100112 (2010)
JN788859.1 Avian coronavirus isolate J1544/Northern Pintail/100112 (2010)
JN788861.1 Avian coronavirus isolate J1573/100112 (2010)
JN788864.1 Avian coronavirus isolate J1616/Northern Pintail/100112 (2010)
JN788868.1 Avian coronavirus isolate K547/Northern Shoveler/091223 (2009)
JN788872.1 Avian coronavirus isolate K570/Northern Shoveler/091223 (2009)
JN788876.1 Avian coronavirus isolate K586/091223 (2009)
JN788877.1 Avian coronavirus isolate K589/Northern Shoveler/091223 (2009)
JN788798.1 Avian coronavirus isolate J0755/Northern Shoveler/091217 (2009)
JN788789.1 Avian coronavirus isolate J0573/Northern Shoveler/091127 (2009)
JN788787.1 Avian coronavirus isolate J0565/Northern Shoveler/091127 (2009)
JN788785.1 Avian coronavirus isolate J0554/Northern Shoveler/091127 (2009)
Pacific Black Duck CoV -G0001-2016/12/21-LC/VIC
Pacific Black Duck CoV-9721-2016/12/22-LC/VIC
Pacific Black Duck CoV-9719-2016/12/22-LC/VIC
Pacific Black Duck CoV-9718-2016/12/22-LC/VIC
Pacific Black Duck CoV-9711-2016/12/21-LC/VIC
Pacific Black Duck CoV-8907-2016/6/07-LC/VIC
Grey Teal CoV-10243-2016/10/29-MM/NSW
Grey Teal CoV-10235-2016/10/28-MM/NSW
18
Grey Teal CoV-10234-2016/10/28-MM/NSW
Grey Teal CoV-10229-2016/10/28-MM/NSW
Grey Teal CoV-10212-2016/6/13-CR/VIC
Grey Teal CoV-10181-2016/6/13-CR/VIC
Pacific Black Duck CoV-10180-2016/6/13-CR/VIC
Grey Teal CoV-10178-2016/6/13-CR/VIC
Grey Teal CoV-10002-2016/6/11-PV/VIC
KF437677.1 Avian coronavirus isolate 12WB69/Spot-billed Duck/2012
JN788863.1 Avian coronavirus isolate J1592/Northern Pintail/100112 (2010)
JN788836.1 Avian coronavirus isolate J1416/Northern Pintail/091230 (2009)
JN788832.1 Avian coronavirus isolate J1396/Northern Pintail/091230 (2009)
63
JN788828.1 Avian coronavirus isolate J1375/Northern Pintail/091223 (2009)
JN788827.1 Avian coronavirus isolate J13645/091223 (2009)
KF437668.1 Avian coronavirus isolate 12WB16/Spot-billed Duck/2012
KF437669.1 Avian coronavirus isolate 12WB18/Spot-billed Duck/2012
46
KF437670.1 Avian coronavirus isolate 12WB19/Spot-billed Duck/2012
44
KF437672.1 Avian coronavirus isolate 12WB52/Spot-billed Duck/2012
KF437674.1 Avian coronavirus isolate 12WB55/Spot-billed Duck/2012
34
KF437667.1 Avian coronavirus isolate 12WB14/Spot-billed Duck/2012
KF437678.1 Avian coronavirus isolate 12WB70/Spot-billed Duck/2012
KF437671.1 Avian coronavirus isolate 12WB49/Spot-billed Duck/2012
29
KF437675.1 Avian coronavirus isolate 12WB62/Spot-billed Duck/2012
KF437679.1 Avian coronavirus isolate 12WB71/Spot-billed Duck/2012
Grey Teal CoV-10250-2016/10/29-MM/NSW
KT222511.1 Duck-dominant coronavirus isolate DdCoV/DK/fujian/90/2013
JN788871.1 Avian coronavirus isolate K561/Northern Shoveler/091223 (2009)
34
JN788848.1 Avian coronavirus isolate J1491/Northern Shoveler/100112 (2010)
KF437676.1 Avian coronavirus isolate 12WB63/Spot-billed Duck/2012
GU396687.1 Rock sandpiper coronavirus CIR-65824 (2005)
27
GU396688.1 Rock sandpiper coronavirus CIR-65828 (2005)
GU396689.1 Rock sandpiper coronavirus CIR-65885 (2005)
KP033064.1 Duck-dominant coronavirus isolate DdCoV/DK/Guangdong/F07/2014
KM093873.1 Avian coronavirus isolate D11/White-faced Whistling Duck/2011
KM093874.1 Avian coronavirus isolate D15/Red-billed Teal/2011
35
KM093872.1 Avian coronavirus isolate D9/White-faced Whistling Duck/2011
KT222512.1 Duck-dominant coronavirus isolate DdCoV/DK/fujian/64/2013
KT222523.1 Duck-dominant coronavirus isolate DdCoV/DK/fujian/63/2013
KM093875.1 Avian coronavirus isolate D10/Red-billed Teal/2011
62
KT222533.1 Duck-dominant coronavirus isolate DdCoV/DK/hubei/FR225/2013
KT222534.1 Duck-dominant coronavirus isolate DdCoV/DK/hubei/FR226/2013
JN788780.1 Avian coronavirus isolate J0109/Eurasian Teal/091106 (2009)
JN788781.1 Avian coronavirus isolate J0121/Eurasian Teal/091106 (2009)
JN788782.1 Avian coronavirus isolate J0122/Eurasian Teal/091106 (2009)
JN788783.1 Avian coronavirus isolate J0124/091106 (2009)
26
JN788784.1 Avian coronavirus isolate J0126/Eurasian Teal/091106 (2009)
JN788795.1 Avian coronavirus isolate J0589/Eurasian Teal/091127 (2009)
JN788847.1 Avian coronavirus isolate J1482/Tufted Duck/100112 (2010)
KY320400.1 Avian coronavirus isolate 271/Mallard Duck/Sweden/2014
KY320402.1 Avian coronavirus isolate 665/Mallard Duck/Sweden/2014
KM093876.1 Avian coronavirus isolate D13/Red-billed Teal/2011
KM093877.1 Avian coronavirus isolate D12/Red-billed Teal/2011
KM093878.1 Avian coronavirus isolate D16/White-faced Whistling Duck/2011
Grey Teal CoV-10228-2016/10/28-MM/NSW
KP033048.1 Duck-dominant coronavirus isolate DdCoV/DK/Guangdong/F93/2014
62
KP033059.1 Duck-dominant coronavirus isolate DdCoV/DK/Guangdong/F31/2014
KP033038.1 Duck-dominant coronavirus isolate DdCoV/DK/Guangdong/H1373/2014
KF437666.1 Avian coronavirus isolate 12WB180/Pintail/2012
JN788794.1 Avian coronavirus isolate J0588/Eurasian Wigeon/091127 (2009)
KM454473.1 Duck coronavirus isolate DK/GD/27/2014
Pacific Black Duck CoV-10196-2016/6/13-CR/VIC
Grey Teal CoV-10214-2016/6/13-CR/VIC
Grey Teal CoV-10230-2016/10/28-MM/NSW
Radjah Shelduck CoV-9515-2016/4/28-HD/NT
Ruddy Turnstone CoV-9564-2016/11/19-BB/TAS
Pacific Black Duck CoV-9710-2016/12/21-LC/VIC
Curlew Sandpiper CoV-9776-2016/12/28-WS/VIC
Curlew Sandpiper CoV-9819-2016/12/30-WS/VIC
Curlew Sandpiper CoV-9822-2016/12/30-WS/VIC
GU396668.1 Pintail coronavirus PB/AK-10 (2005)
GU396671.1 Pintail coronavirus PB/AK-25 (2005)
GU396672.1 Pintail coronavirus PB/AK-37 (2005)
GU396673.1 Pintail coronavirus PB/AK-124 (2005)
JN788786.1 Avian coronavirus isolate J0559/Eurasian Teal/091127 (2009)
JN788790.1 Avian coronavirus isolate J0576/Eurasian Wigeon/091127(2009)
JN788792.1 Avian coronavirus isolate J0579/Eurasian Teal/091127 (2009)
JN788878.1 Avian coronavirus isolate K596/Eurasian Wigeon/091223 (2009)
JN788873.1 Avian coronavirus isolate K572/Eurasian Wigeon/091223 (2009)
JN788870.1 Avian coronavirus isolate K5556/091223 (2009)
JN788869.1 Avian coronavirus isolate K554/Northern Shoveler/091223 (2009)
JN788866.1 Avian coronavirus isolate K5334/091223 (2009)
JN788862.1 Avian coronavirus isolate J1581/Northern Pintail/100112 (2010)
JN788860.1 Avian coronavirus isolate J1561/Eurasian Wigeon/100112 (2010)
JN788851.1 Avian coronavirus isolate J1502/100112 (2010)
JN788843.1 Avian coronavirus isolate J1471/091230 (2009)
JN788842.1 Avian coronavirus isolate J1470/Eurasian Wigeon/091230 (2009)
JN788833.1 Avian coronavirus isolate J1404/Northern Pintail/091230 (2009)
JN788830.1 Avian coronavirus isolate J1393/Northern Pintail/091230 (2009)
JN788829.1 Avian coronavirus isolate J13834/091230 (2009)
JN788826.1 Avian coronavirus isolate J1362/Northern Shoveler/091223 (2009)
JN788810.1 Avian coronavirus isolate J1042/Eurasian Wigeon/091217 (2009)
JN788808.1 Avian coronavirus isolate J10127/091217 (2009)
JN788799.1 Avian coronavirus isolate J07793/091217 (2009)
KJ741871.1 Avian coronavirus strain OH11 Eurasian Teal (2009)
KJ741878.1 Avian coronavirus strain OH33 Mallard Duck (2009)
KJ741879.1 Avian coronavirus strain OH34 Eurasian Teal (2009)
KJ741881.1 Avian coronavirus strain OH38 Mallard Duck (2009)
KM015306.1 Avian coronavirus isolate Mallard Duck/90A88310/129776/110829 (2011)
KM015307.1 Avian coronavirus isolate Mallard Duck/90A88309/129823/110902 (2011)
KM015308.1 Avian coronavirus isolate Mallard Duck/90A88309/130049/110916 (2011)
KM015309.1 Avian coronavirus isolate Mallard Duck/90A88390/131192/111020 (2011)
KM015310.1 Avian coronavirus isolate Mallard Duck/90A88491/131624/111024 (2011)
KM015312.1 Avian coronavirus isolate Mallard Duck/90A88582/133583/111123 (2011)
KM015313.1 Avian coronavirus isolate Mallard Duck/90A88582/133648/111124 (2011)
KM015314.1 Avian coronavirus isolate Mallard Duck/90A88542/131420/111023 (2011)
KM015315.1 Avian coronavirus isolate Mallard Duck/90A88434/131444/111023 (2011)
KM015316.1 Avian coronavirus isolate Mallard Duck/90A88641/132624/111107 (2011)
KM015317.1 Avian coronavirus isolate Mallard Duck/90A88460/132681/111108 (2011)
KM015318.1 Avian coronavirus isolate Mallard Duck/90A88527/132886/111111 (2011)
KM015319.1 Avian coronavirus isolate Mallard Duck/90A88372/133001/111112 (2011)
KM015320.1 Avian coronavirus isolate Mallard Duck/90A88384/133035/111113 (2011)
KM015321.1 Avian coronavirus isolate Mallard Duck/90A88681/133134/111115 (2011)
KM015322.1 Avian coronavirus isolate Mallard Duck/90A88681/133195/111116 (2011)
KM015323.1 Avian coronavirus isolate Mallard Duck/90A88355/133731/111125 (2011)
KM015324.1 Avian coronavirus isolate Mallard Duck/90A88310/132926/111111 (2011)
KM015325.1 Avian coronavirus isolate Mallard Duck/90A88548/133595/111123 (2011)
KM015326.1 Avian coronavirus isolate Mallard Duck/90A88566/133629/111124 (2011)
KP033039.1 Duck-dominant coronavirus isolate DdCoV/DK/Guangdong/H1001-2/2014
KP033040.1 Duck-dominant coronavirus isolate DdCoV/DK/Guangdong/H1001-1/2014
KP033043.1 Duck-dominant coronavirus isolate DdCoV/DK/Zhejiang/1411/2014
KP033056.1 Duck-dominant coronavirus isolate DdCoV/DK/Guangdong/F34/2014
KP033060.1 Duck-dominant coronavirus isolate DdCoV/DK/Guangdong/F28/2014
KP033062.1 Duck-dominant coronavirus isolate DdCoV/DK/Guangdong/F11/2014
KP033070.1 Duck-dominant coronavirus isolate DdCoV/DK/Anhui/F82/2014
KP033078.1 Duck-dominant coronavirus isolate DdCoV/DK/Anhui/1656/2014
KT222497.1 Duck-dominant coronavirus isolate DdCoV/DK/anhui/FA271/2013
KT222498.1 Duck-dominant coronavirus isolate DdCoV/DK/guizhou/FZ253/2013
KT222499.1 Duck-dominant coronavirus isolate DdCoV/DK/guizhou/FZ252/2013
KT222500.1 Duck-dominant coronavirus isolate DdCoV/DK/sichuan/FP277/2013
KT222501.1 Duck-dominant coronavirus isolate DdCoV/DK/guizhou/FZ251/2013
KT222502.1 Duck-dominant coronavirus isolate DdCoV/DK/sichuan/HP230/2013
KT222503.1 Duck-dominant coronavirus isolate DdCoV/DK/sichuan/FP219/2013
KT222508.1 Duck-dominant coronavirus isolate DdCoV/DK/sichuan/FP212/2013
KT222509.1 Duck-dominant coronavirus isolate DdCoV/DK/guizhou/FZ254/2013
KT222513.1 Duck-dominant coronavirus isolate DdCoV/DK/fujian/67/2013
KT222514.1 Duck-dominant coronavirus isolate DdCoV/DK/sichuan/HP229/2013
KT222515.1 Duck-dominant coronavirus isolate DdCoV/DK/sichuan/FP247/2013
KT222517.1 Duck-dominant coronavirus isolate DdCoV/DK/sichuan/FP244/2013
KT222518.1 Duck-dominant coronavirus isolate DdCoV/DK/sichuan/FP248/2013
KT222519.1 Duck-dominant coronavirus isolate DdCoV/DK/sichuan/FP246/2013
KT222520.1 Duck-dominant coronavirus isolate DdCoV/DK/sichuan/FP243/2013
KT222521.1 Duck-dominant coronavirus isolate DdCoV/DK/sichuan/FP242/2013
KT222522.1 Duck-dominant coronavirus isolate DdCoV/DK/sichuan/FP241/2013
KT222525.1 Duck-dominant coronavirus isolate DdCoV/DK/sichuan/FP276/2013
KT222529.1 Duck-dominant coronavirus isolate DdCoV/DK/hubei/FR228/2013
KT222530.1 Duck-dominant coronavirus isolate DdCoV/DK/hubei/FR266/2013
KT222535.1 Duck-dominant coronavirus isolate DdCoV/DK/hubei/FR217/2013
KT222536.1 Duck-dominant coronavirus isolate DdCoV/DK/jiangsu/FK278/2013
KT222537.1 Duck-dominant coronavirus isolate DdCoV/DK/anhui/FA246/2013
KT222540.1 Duck-dominant coronavirus isolate DdCoV/DK/hubei/FR215/2013
KT222544.1 Duck-dominant coronavirus isolate DdCoV/Pigeon/guangdong/FY253/2013
KT254275.1 Duck-dominant coronavirus isolate DdCoV/DK/Guangdong/F11/2014
KT882616.1 Avian coronavirus isolate AvCoV/Greater Scaup/67699/2007
KT882626.1 Avian coronavirus isolate AvCoV/Mallard/70046/2007
KT882628.1 Avian coronavirus isolate AvCoV/Mallard/70125/2007
KY320401.1 Avian coronavirus isolate 485/Mallard Duck/Sweden/2014
KY320404.1 Avian coronavirus isolate 937/Mallard Duck/Sweden/2015
KM093879.1 Avian coronavirus isolate S22/Kittlitzs Plover/2011
KM093880.1 Avian coronavirus isolate S21/Hottentot Teal/2011
KM093881.1 Avian coronavirus isolate S23/Common Moorhen/2011
KM093882.1 Avian coronavirus isolate 1P/Greater Painted Snipe/2011
KM093883.1 Avian coronavirus isolate 2P/Kittlitzs Plover/2011
KM093884.1 Avian coronavirus isolate 3P/Kittlitzs Plover/2011
KM093885.1 Avian coronavirus isolate 4P/Common Moorhen/2011
KM093886.1 Avian coronavirus isolate 7V/Red Fody/2011
KM093893.1 Avian coronavirus isolate CV11/Allens Gallinule/2011
KM093896.1 Avian coronavirus isolate 5cvPF9/Madagascan Rail/2011
KM093899.1 Avian coronavirus isolate 9cvPF17/Western Cattle Egret/2011
KM093895.1 Avian coronavirus isolate 4cvPF7/Madagascan Snipe/2011
KM093897.1 Avian coronavirus isolate 6cvPF12/Western Cattle Egret/2011
Red-necked Stint CoV-9772-2016/12/28-WS/VIC
Red-necked Stint CoV-9786-2016/12/29-WS/VIC
65
Red-necked Stint CoV-9752-2016/12/28-WS/VIC
Red-necked Stint CoV-9738-2016/12/28-WS/VIC
Red-necked Stint CoV-9729-2016/12/28-WS/VIC
Red-necked Stint CoV-10402-2017/1/21-BI/VIC
Red-necked Stint CoV-10415-2017/1/21-BI/VIC
Red-necked Stint CoV-9068-2016/12/30-WS/VIC
Ruddy Turnstone CoV-9596-2016/11/19-CM/TAS
Ruddy Turnstone CoV-9566-2016/11/18-BB/TAS
Ruddy Turnstone CoV-9592-2016/11/18-BB/TAS
Ruddy Turnstone CoV-9612-2016/11/19-CM/TAS
Ruddy Turnstone CoV-9617-2016/11/19-CM/TAS
Ruddy Turnstone CoV-9624-2016/11/19-CM/TAS
Ruddy Turnstone CoV-9628-2016/11/22-DW/TAS
62
Ruddy Turnstone CoV-9662-2016/11/27-NV/SA
Ruddy Turnstone CoV-9694-2016/11/28-BP/SA
Ruddy Turnstone CoV-9696-2016/11/28-BP/SA
GU396674.1 Glaucus-gull coronavirus PB/AK-173 (2005)
GU396679.1 Black-headed gull coronavirus CIR-66183 (2005)
GU396680.1 Black-headed gull coronavirus CIR-66185 (2005)
GU396681.1 Black-headed gull coronavirus CIR-66187 (2005)
GU396682.1 Glaucous-winged gull CIR-66002 (2005)
GU396683.1 Black-headed gull coronavirus CIR-66144 (2005)
GU396684.1 Black-headed gull coronavirus CIR-66146 (2005)
GU396686.1 Black-headed gull coronavirus CIR-66152 (2005)
0.005

Supplementary Figure S3. *Maximum likelihood phylogenetic tree of the 311 nt fragment of the polymerase gene of deltacoronaviruses from wild birds, including the sequences obtained in this study. Bootstrap confidence of each branch was calculated from 1000 replicates. The sampling location and state of each Australian sample is identified with a two letter code and the state: New South Wales (NSW): MM-Moulamein; Northern Territory (NT): HD-Humpty Doo; South Australia (SA): BP-Boatswain Point, NV-Nene Valley; Tasmania (TAS): BB-Borges Bay, King Island, CM-Central Manuka, King Island, DW-Dripping Wells, King Island, Tasmania; Victoria (VIC): BI-Barrallier Island, CR-Carlisle River, LC-Lake Connewarre, PV-Paynesville, WS-Werribee South.*

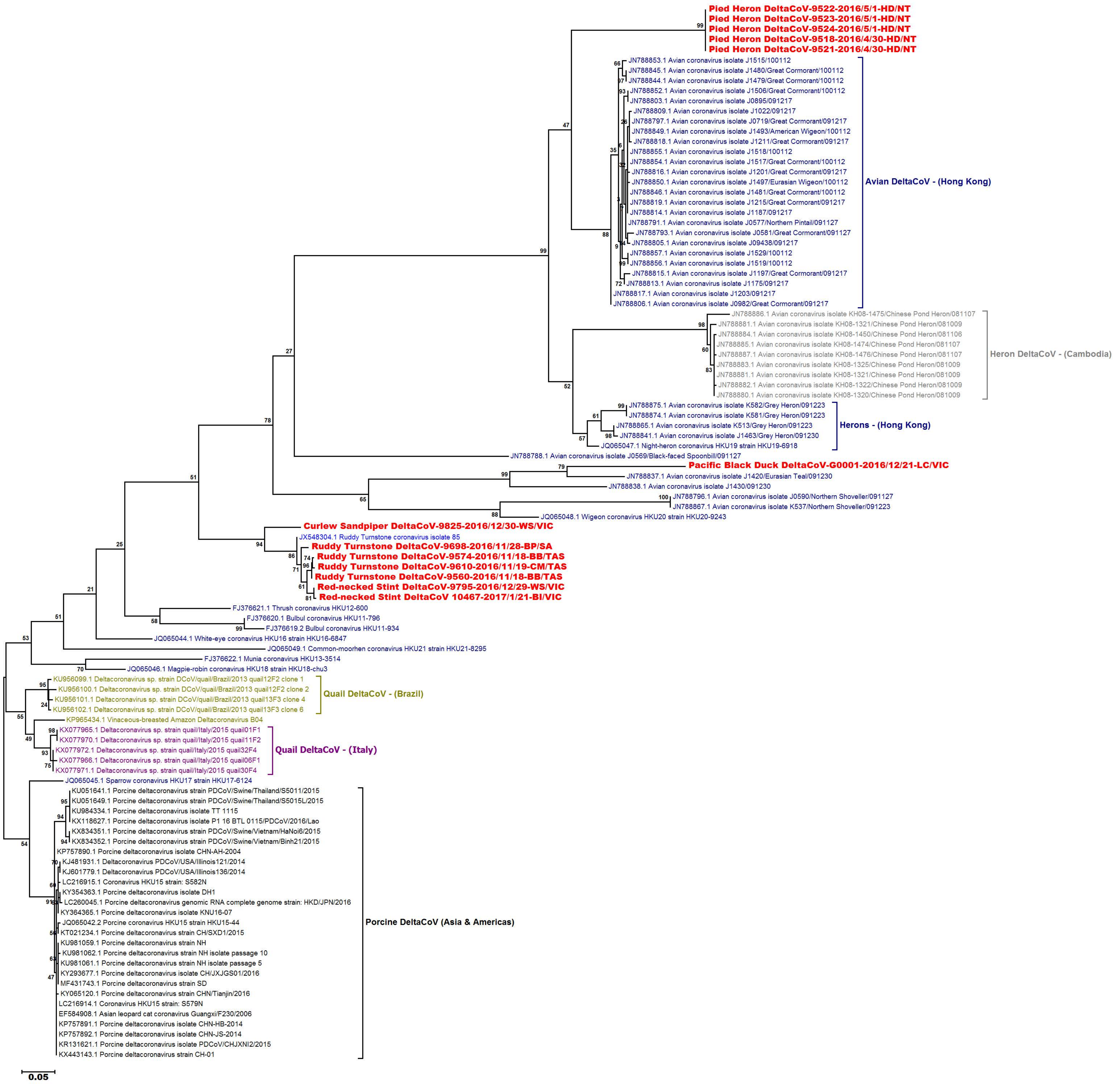

Pied Heron DeltaCoV-9522-2016/5/1-HD/NT
Pied Heron DeltaCoV-9523-2016/5/1-HD/NT
Pied Heron DeltaCoV-9524-2016/5/1-HD/NT
Pied Heron DeltaCoV-9518-2016/4/30-HD/NT
Pied Heron DeltaCoV-9521-2016/4/30-HD/NT
JN788853.1 Avian coronavirus isolate J1515/100112
JN788845.1 Avian coronavirus isolate J1480/Great Cormorant/100112
JN788844.1 Avian coronavirus isolate J1479/Great Cormorant/100112
JN788852.1 Avian coronavirus isolate J1506/Great Cormorant/100112
JN788803.1 Avian coronavirus isolate J0895/091217
JN788809.1 Avian coronavirus isolate J1022/091217
JN788797.1 Avian coronavirus isolate J0719/Great Cormorant/091217
JN788849.1 Avian coronavirus isolate J1493/American Wigeon/100112
JN788818.1 Avian coronavirus isolate J1211/Great Cormorant/091217
JN788855.1 Avian coronavirus isolate J1518/100112
JN788854.1 Avian coronavirus isolate J1517/Great Cormorant/100112
JN788816.1 Avian coronavirus isolate J1201/Great Cormorant/091217
JN788850.1 Avian coronavirus isolate J1497/Eurasian Wigeon/100112
JN788846.1 Avian coronavirus isolate J1481/Great Cormorant/100112
JN788819.1 Avian coronavirus isolate J1215/Great Cormorant/091217
JN788814.1 Avian coronavirus isolate J1187/091217
JN788791.1 Avian coronavirus isolate J0577/Northern Pintail/091127
JN788793.1 Avian coronavirus isolate J0581/Great Cormorant/091127
JN788805.1 Avian coronavirus isolate J09438/091217
JN788857.1 Avian coronavirus isolate J1529/100112
JN788856.1 Avian coronavirus isolate J1519/100112
JN788815.1 Avian coronavirus isolate J1197/Great Cormorant/091217
JN788813.1 Avian coronavirus isolate J1175/091217
JN788817.1 Avian coronavirus isolate J1203/091217
JN788806.1 Avian coronavirus isolate J0982/Great Cormorant/091217
Avian DeltaCoV - (Hong Kong)
JN788886.1 Avian coronavirus isolate KH08-1475/Chinese Pond Heron/081107
JN788881.1 Avian coronavirus isolate KH08-1321/Chinese Pond Heron/081009
JN788884.1 Avian coronavirus isolate KH08-1450/Chinese Pond Heron/081106
JN788885.1 Avian coronavirus isolate KH08-1474/Chinese Pond Heron/081107
JN788887.1 Avian coronavirus isolate KH08-1476/Chinese Pond Heron/081107
JN788883.1 Avian coronavirus isolate KH08-1325/Chinese Pond Heron/081009
JN788881.1 Avian coronavirus isolate KH08-1321/Chinese Pond Heron/081009
JN788882.1 Avian coronavirus isolate KH08-1322/Chinese Pond Heron/081009
JN788880.1 Avian coronavirus isolate KH08-1320/Chinese Pond Heron/081009
Heron DeltaCoV - (Cambodia)
JN788875.1 Avian coronavirus isolate K582/Grey Heron/091223
JN788874.1 Avian coronavirus isolate K581/Grey Heron/091223
JN788865.1 Avian coronavirus isolate K513/Grey Heron/091223
JN788841.1 Avian coronavirus isolate J1463/Grey Heron/091230
JQ065047.1 Night-heron coronavirus HKU19 strain HKU19-6918
Herons - (Hong Kong)
JN788788.1 Avian coronavirus isolate J0569/Black-faced Spoonbill/091127
Pacific Black Duck DeltaCoV-G0001-2016/12/21-LC/VIC
JN788837.1 Avian coronavirus isolate J1420/Eurasian Teal/091230
JN788838.1 Avian coronavirus isolate J1430/091230
JN788796.1 Avian coronavirus isolate J0590/Northern Shoveller/091127
JN788867.1 Avian coronavirus isolate K537/Northern Shoveller/091223
JQ065048.1 Wigeon coronavirus HKU20 strain HKU20-9243
Curlew Sandpiper DeltaCoV-9825-2016/12/30-WS/VIC
JX548304.1 Ruddy Turnstone coronavirus isolate 85
Ruddy Turnstone DeltaCoV-9698-2016/11/28-BP/SA
Ruddy Turnstone DeltaCoV-9574-2016/11/18-BB/TAS
Ruddy Turnstone DeltaCoV-9610-2016/11/19-CM/TAS
Ruddy Turnstone DeltaCoV-9560-2016/11/18-BB/TAS
Red-necked Stint DeltaCoV-9795-2016/12/29-WS/VIC
Red-necked Stint DeltaCoV 10467-2017/1/21-BI/VIC
FJ376621.1 Thrush coronavirus HKU12-600
FJ376620.1 Bulbul coronavirus HKU11-796
FJ376619.2 Bulbul coronavirus HKU11-934
JQ065044.1 White-eye coronavirus HKU16 strain HKU16-6847
JQ065049.1 Common-moorhen coronavirus HKU21 strain HKU21-8295
FJ376622.1 Munia coronavirus HKU13-3514
JQ065046.1 Magpie-robin coronavirus HKU18 strain HKU18-chu3
KU956099.1 Deltacoronavirus sp. strain DCoV/quail/Brazil/2013 quail12F2 clone 1
KU956100.1 Deltacoronavirus sp. strain DCoV/quail/Brazil/2013 quail12F2 clone 2
KU956101.1 Deltacoronavirus sp. strain DCoV/quail/Brazil/2013 quail13F3 clone 4
KU956102.1 Deltacoronavirus sp. strain DCoV/quail/Brazil/2013 quail13F3 clone 6
Quail DeltaCoV - (Brazil)
KP965434.1 Vinaceous-breasted Amazon Deltacoronavirus B04
KX077965.1 Deltacoronavirus sp. strain quail/Italy/2015 quail01F1
KX077970.1 Deltacoronavirus sp. strain quail/Italy/2015 quail11F2
KX077972.1 Deltacoronavirus sp. strain quail/Italy/2015 quail32F4
KX077966.1 Deltacoronavirus sp. strain quail/Italy/2015 quail06F1
KX077971.1 Deltacoronavirus sp. strain quail/Italy/2015 quail30F4
Quail DeltaCoV - (Italy)
JQ065045.1 Sparrow coronavirus HKU17 strain HKU17-6124
KU051641.1 Porcine deltacoronavirus strain PDCoV/Swine/Thailand/S5011/2015
KU051649.1 Porcine deltacoronavirus strain PDCoV/Swine/Thailand/S5015L/2015
KU984334.1 Porcine deltacoronavirus isolate TT 1115
KX118627.1 Porcine deltacoronavirus isolate P1 16 BTL 0115/PDCoV/2016/Lao
KX834351.1 Porcine deltacoronavirus strain PDCoV/Swine/Vietnam/HaNoi6/2015
KX834352.1 Porcine deltacoronavirus strain PDCoV/Swine/Vietnam/Binh21/2015
KP757890.1 Porcine deltacoronavirus isolate CHN-AH-2004
KJ481931.1 Deltacoronavirus PDCoV/USA/Illinois121/2014
KJ601779.1 Deltacoronavirus PDCoV/USA/Illinois136/2014
LC216915.1 Coronavirus HKU15 strain: S582N
KY354363.1 Porcine deltacoronavirus isolate DH1
LC260045.1 Porcine deltacoronavirus genomic RNA complete genome strain: HKD/JPN/2016
KY364365.1 Porcine deltacoronavirus isolate KNU16-07
JQ065042.2 Porcine coronavirus HKU15 strain HKU15-44
KT021234.1 Porcine deltacoronavirus strain CH/SXD1/2015
KU981059.1 Porcine deltacoronavirus strain NH
KU981062.1 Porcine deltacoronavirus strain NH isolate passage 10
KU981061.1 Porcine deltacoronavirus strain NH isolate passage 5
KY293677.1 Porcine deltacoronavirus isolate CH/JXJGS01/2016
MF431743.1 Porcine deltacoronavirus strain SD
KY065120.1 Porcine deltacoronavirus strain CHN/Tianjin/2016
LC216914.1 Coronavirus HKU15 strain: S579N
EF584908.1 Asian leopard cat coronavirus Guangxi/F230/2006
KP757891.1 Porcine deltacoronavirus isolate CHN-HB-2014
KP757892.1 Porcine deltacoronavirus isolate CHN-JS-2014
KR131621.1 Porcine deltacoronavirus isolate PDCoV/CHJXNI2/2015
KX443143.1 Porcine deltacoronavirus strain CH-01
Porcine DeltaCoV (Asia & Americas)
0.05
